# The CALCINEURIN B-LIKE 4/CBL-INTERACTING PROTEIN 3 module degrades repressor JAZ5 during rose petal senescence

**DOI:** 10.1093/plphys/kiad365

**Published:** 2023-07-04

**Authors:** Changxi Chen, Yanxing Ma, Lanxin Zuo, Yue Xiao, Yunhe Jiang, Junping Gao

**Affiliations:** Beijing Key Laboratory of Development and Quality Control of Ornamental Crops, Department of Ornamental Horticulture, College of Horticulture, China Agricultural University, Beijing 100193, China; Beijing Key Laboratory of Development and Quality Control of Ornamental Crops, Department of Ornamental Horticulture, College of Horticulture, China Agricultural University, Beijing 100193, China; Beijing Key Laboratory of Development and Quality Control of Ornamental Crops, Department of Ornamental Horticulture, College of Horticulture, China Agricultural University, Beijing 100193, China; Beijing Key Laboratory of Development and Quality Control of Ornamental Crops, Department of Ornamental Horticulture, College of Horticulture, China Agricultural University, Beijing 100193, China; Beijing Key Laboratory of Development and Quality Control of Ornamental Crops, Department of Ornamental Horticulture, College of Horticulture, China Agricultural University, Beijing 100193, China; Beijing Key Laboratory of Development and Quality Control of Ornamental Crops, Department of Ornamental Horticulture, College of Horticulture, China Agricultural University, Beijing 100193, China

## Abstract

Flower senescence is genetically regulated and developmentally controlled. The phytohormone ethylene induces flower senescence in rose (*Rosa hybrida*), but the underlying signaling network is not well understood. Given that calcium regulates senescence in animals and plants, we explored the role of calcium in petal senescence. Here, we report that the expression of *calcineurin B-like protein 4* (*RhCBL4*), which encodes a calcium receptor, is induced by senescence and ethylene signaling in rose petals. RhCBL4 interacts with CBL-interacting protein kinase 3 (RhCIPK3), and both positively regulate petal senescence. Furthermore, we determined that RhCIPK3 interacts with the jasmonic acid response repressor jasmonate ZIM-domain 5 (RhJAZ5). RhCIPK3 phosphorylates RhJAZ5 and promotes its degradation in the presence of ethylene. Our results reveal that the RhCBL4-RhCIPK3-RhJAZ5 module mediates ethylene-regulated petal senescence. These findings provide insights into flower senescence, which may facilitate innovations in postharvest technology for extending rose flower longevity.

## Teaser

The CBL4-CIPK3 module degrades the jasmonic acid response repressor JAZ5 in response to ethylene to regulate petal senescence in rose.

## Introduction

Flower senescence, the last event in floral development, is characterized by fading, wilting, and abscission (Ma et al. 2018). These changes are genetically regulated and developmentally controlled (van Doorn and Woltering 2008). Petal senescence is a key factor affecting the quality of ornamental plants and is controlled by a combination of gene expression and phytohormone signaling (Rogers 2013; Ma et al. 2018). Changes in gene expression during flower senescence have been analyzed using microarrays from different plant species (van Doorn et al. 2003; Breeze et al. 2004; Price et al. 2008; Wagstaff et al. 2010). A group of marker genes known as senescence-associated genes (SAGs), including genes encoding certain catabolic enzymes and transcription factors (TFs), are upregulated during senescence (Lim et al. 2007; Guo and Gan 2012).

Changes in endogenous phytohormone levels influence signaling networks that function in senescence processes (van Doorn and Woltering 2008; Zhang, Guo, et al. 2021). Ethylene signaling transduction is well studied in *Arabidopsis* (*Arabidopsis thaliana*) (Wang and Qiao 2019; Zhao et al. 2021). Ethylene binds its receptors, leading to inactivation of Constitutive Triple Response 1 (CTR1), resulting in the unphosphorylated Ethylene Insensitive 2 (EIN2) to be cleaved and transferred into nucleus (Kieber et al. 1993; Alonso et al. 1999; Bleecker and Kende 2000; Ju et al. 2012). In the nucleus, EIN2 stabilizes the TFs EIN3/EIN3-Like 1 (EIL1), in-turn promoting the expression of ethylene-responsive genes (Chao et al. 1997; Qiao et al. 2012). It is well known that ethylene acts as a master regulator of flower senescence (Rogers 2013; Ma et al. 2018). In ethylene-sensitive flowers, such as rose, senescence is accompanied by increased ethylene production (Ma et al. 2005), and ethylene affects both the opening and senescence of rose flowers (Ma et al. 2006; Cheng et al. 2021). Exogenous ethylene treatment further accelerates ethylene production, petal wilting, and abscission (Ma et al. 2005, 2018; Shibuya et al. 2013).

Jasmonic acid (JA) signal pathway is also well established in *Arabidopsis* (Chini et al. 2016; Howe et al. 2018). JASMONATE ZIM-domain (JAZ) proteins are key suppressors in JA signal pathway (Chini et al. 2007; Pauwels and Goossens 2011). In basal condition, JAZs recruit corepressor to repress downstream TFs (Howe et al. 2018). Accumulation of JA triggers JAZ proteins to bind Coronatine Insensitive 1 (COI1), resulting in ubiquitination and 26S proteasomal degradation of JAZs, and then activates downstream TFs (Chini et al. 2007, 2016; Thines et al. 2007; Howe et al. 2018). Regarding the role of JA in petal senescence, it is reported that the applications of JA accelerated petal senescence in petunia (*Petunia hybrida*), orchid (*Dendrobium hybrida*), and rose (*Rosa hybrida*) (Porat et al. 1993; Zhang, Zhao, et al. 2019). Recent research has found that JAZ proteins are integral to the accumulation of JA in petal-specific senescence (Serrano-Bueno et al. 2022). Although ethylene and JA signaling pathways have been extensively studied, their crosstalk during petal senescence remains largely unknown.

Petal senescence occurs along with global changes in gene expression (Shibuya 2018; Wojciechowska et al. 2018), and studies in rose have placed much emphasis on identifying regulators, especially TFs, that control this process (Lü et al. 2014; Khaskheli et al. 2018; Zhang, Zhao, et al. 2019). However, the signaling mechanisms of petal senescence remain poorly understood. Calcium (Ca^2+^), a universal second messenger molecule, is prevalent in eukaryotes and is involved in almost every aspect of plant growth and development (Dodd et al. 2010; Spalding and Harper 2011). Extracellular signals, such as hormonal and environmental signals, cause specific changes in Ca^2+^ concentration in cells (Whalley and Knight 2013). These changes in plants are sensed and decoded by a series of calcium receptors and transmitted to downstream target proteins, resulting in a series of biochemical reactions in the cells (Kudla et al. 2010). The calcium receptors in plants identified to date are divided into 3 categories: calmodulin (CaM) and CaM-like proteins (CMLs), calcium-dependent protein kinases (CDPKs/CRKs), and calcineurin B-like proteins (CBLs) (Edel et al. 2017; Tang et al. 2020).

Previous studies have demonstrated that CaMs and CDPKs are involved in leaf senescence (Yang et al. 2022). For example, CaM1 positively regulates receptor-like protein kinase 1 (RPK1)-mediated leaf senescence in *Arabidopsis* (Dai et al. 2018). Calcium-dependent protein kinase 12 (CPK12) involves in leaf senescence by regulating reactive oxygen species and photosynthetic rate in rice (*Oryza sativa*) (Wang et al. 2019). CBLs are a family of small plant-specific proteins (Kudla et al. 2010). CBLs typically bind to CBL-interacting protein kinases (CIPKs), a specific family of proteins in plant signal transduction pathways (Kolukisaoglu et al. 2004). Since the CBL-CIPK signaling is a key regulatory node in controlling various membrane transport processes, the signaling network formed by multiple CBL-CIPK modules has become a major area of focus in the field of plant stress (Tang et al. 2020). The salt overly sensitive (SOS) signaling pathway is the most typical CBL-CIPK module involved in salt stress (Tang et al. 2020). SALT OVERLY SENSITIVE 3 (SOS3/CBL4) and SOS2 (CIPK24) activate the downstream Na^+^ antiporter SOS1 to reduce damage from salt stress in *Arabidopsis* (Qiu et al. 2002). Additionally, AtCIPK14 negatively regulates leaf senescence by phosphorylating WHIRLY1 (WHY1) (Ren et al. 2017). However, it remains unclear whether CBL-CIPK complexes involved in rose petal senescence.

In this study, we found that *RhCBL4* is expressed at high levels in rose during petal senescence and is responsive to ethylene treatment. We demonstrated that the RhCBL4-RhCIPK3 module serves as a positive regulator of petal senescence. We propose that RhCIPK3 regulates petal senescence by interacting with RhJAZ5 and modulating its stability. Our findings provide insights that should be useful for fine-tuning flower senescence in rose.

## Results

### Screening for vital ethylene-related regulators of petal senescence

**Figure 1.**
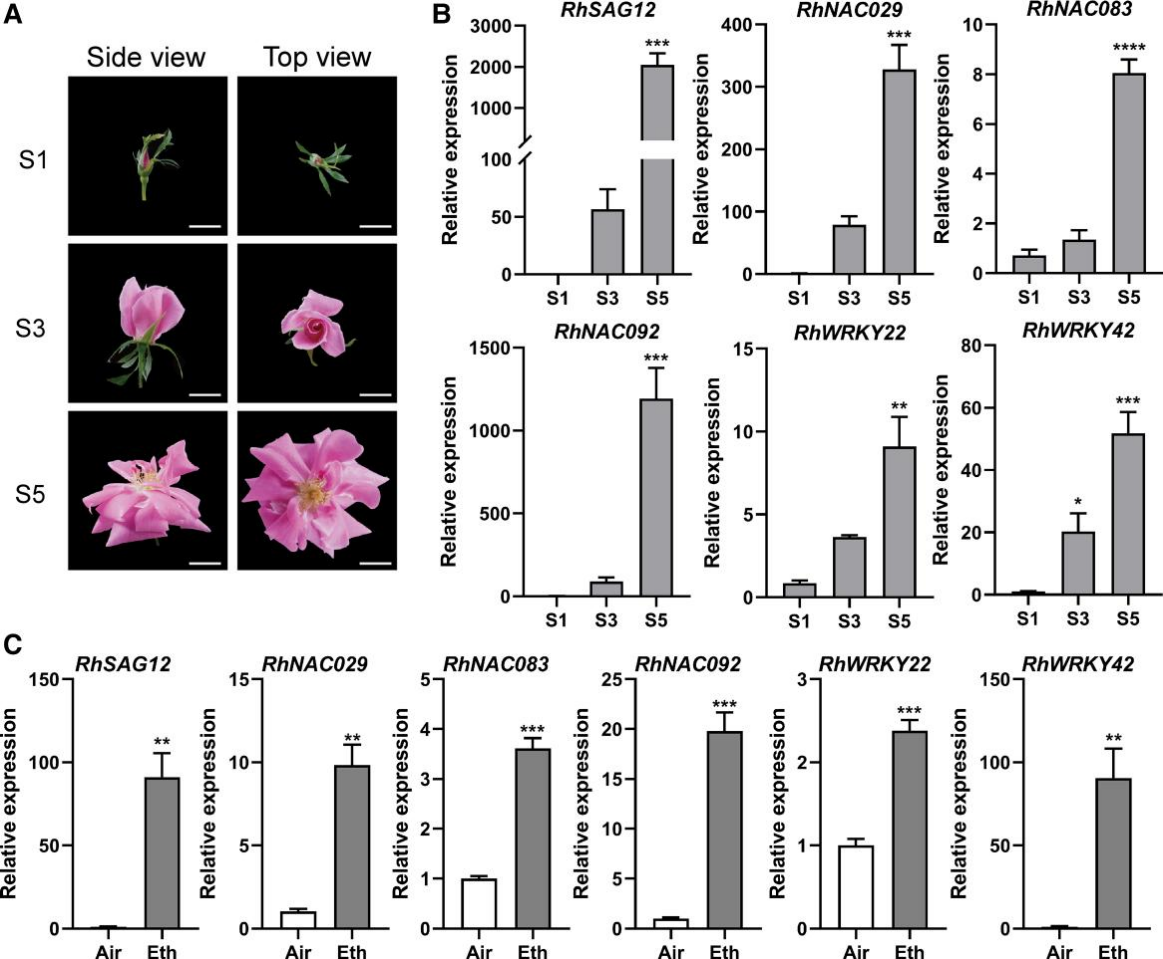
Senescence marker gene expression is induced by aging and ethylene. **A)** Different flower developmental stages of rose flowers. Stage 1 (S1), bud with partially visible petals; Stage 3 (S3), flower with loose outer petals; Stage 5 (S5), fully opened flower. Images were digitally extracted for comparison. Scale bar represents 2 cm. **B)** Relative expression levels of 6 *SAG*s at different stages. **C)** Relative expression levels of 6 *SAG*s with or without 12-h ethylene treatment. For **B)** and **C)**, *RheIF5A* and *RhUBI2* were quantified as internal controls. Each value represents mean ± Sd (Student's *t* test, *n* = 3, *P < 0.05, **P < 0.01, ***P < 0.001, and ****P < 0.0001).

Petal senescence is a unique and highly programmed process that occurs before morphological changes to the flowers are visible (Ma et al. 2018). The rose opening process is divided into 6 stages (Supplemental Fig. S1A) (Ma et al. 2005). To determine the senescence phase of petals, we collected samples at 3 typical stages of flower opening: ready-to-open buds (Stage 1), partially opened flowers (Stage 3), and fully opened flowers (Stage 5) (Fig. 1A). We evaluated the transcript abundance of SAGs at these 3 developmental stages.

Several SAGs, including *RhSAG12*, *RhNAC029*, *RhNAC083*, *RhNAC092*, *RhWRKY22*, and *RhWRKY42*, were significantly upregulated from Stages 1 to 5, especially from Stages 3 to 5 (Fig. 1B). Ethylene was shown to play a vital role in regulating petal senescence (Ma et al. 2005; Ichimura et al. 2009). And ethylene production of rose flower was relatively low at early opening stages and then substantially increased from Stage 3 (Supplemental Fig. S1B). To examine the role of ethylene in rose petal senescence, the rose flowers were treated with exogenous ethylene. Indeed, the transcription of the above SAGs in petals was induced by ethylene treatment (Fig. 1C).

To identify critical regulatory genes involved in petal senescence, we performed transcriptome sequencing to dissect the transcriptomic changes in rose flowers at 3 stages of development and following treatment with ethylene (Jia et al. 2022). We identified differentially expressed genes (DEGs) that were significantly upregulated from Stages 1 to 5 and after ethylene treatment. We identified 3,659, 3,791, and 4,091 DEGs that were significantly upregulated from Stage 3 to Stage 1 (S3 vs. S1), Stage 5 to Stage 1 (S5 vs. S1), and Stage 5 to Stage 3 (S5 vs. S3), respectively (Supplemental Fig. S2 and Table S1). Among these, 409 DEGs were commonly upregulated during all 3 stages of development. In addition, 2,330 DEGs were upregulated after ethylene treatment (Supplemental Fig. S2 and Table S1), and 96 DEGs were commonly upregulated under all conditions (Supplemental Fig. S2 and Table S2). Among these 96 DEGs, several encode TFs and signal transduction components. The Unigene *RchiOBHmChr1g0380691*, encoding a protein belonging to the CBL family, caught our attention. CBL family proteins are calcium sensors required for phytohormone signal transduction and plant developmental processes (Tang et al. 2020). *RchiOBHmChr1g0380691* expression increased during flower senescence and after ethylene treatment in our transcriptome (Supplemental Table S2), suggesting that RhCBL4 may be a candidate related to petal senescence.

### Silencing *RhCBL4* delays petal senescence

**Figure 2.**
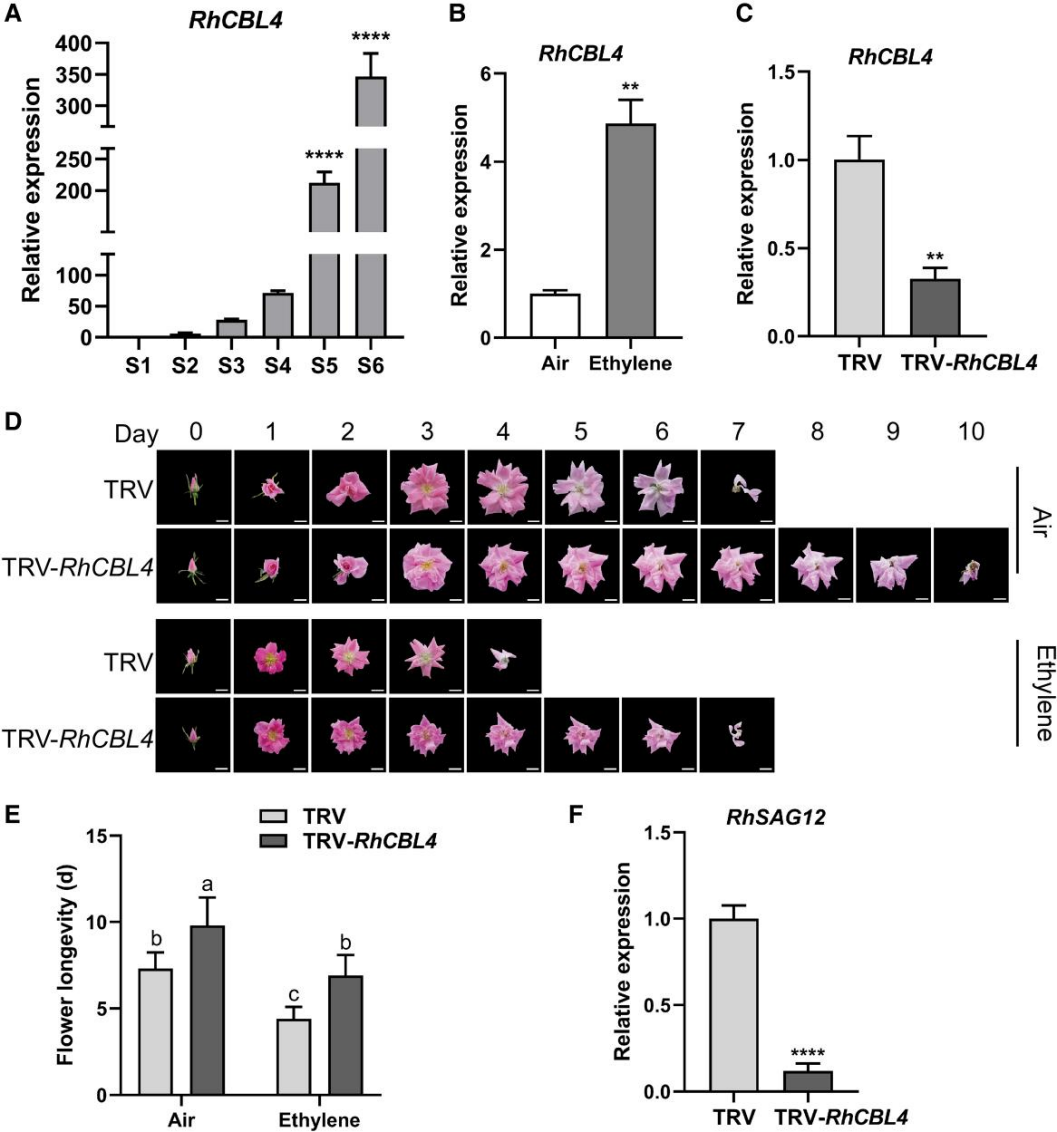
Silencing of *RhCBL4* delays petal senescence in rose. **A)** The RT-qPCR analysis of *RhCBL4* expression in the petal during rose flower opening stages. S, stage. **B)** Expression of *RhCBL4* transcript level in petals under 12-h ethylene treatment. **C)** Expression of *RhCBL4* in the petals of TRV control plants and *RhCBL4*-silenced plants. **D)** Phenotypes of TRV control and *RhCBL4*-silenced flowers with air or ethylene treatment were recorded daily. Images were digitally extracted for comparison. Scale bar represents 2 cm. **E)** The duration of flower senescence in control and *RhCBL4*-silenced flowers (mean ± Sd, *n* = 10, *P* < 0.05, 2-way ANOVA). **F)** Relative expression levels of *RhSAG12* in control and *RhCBL4*-silenced petals by RT-qPCR. For **A)** to **C)** and **F)**, *RheIF5A* and *RhUBI2* were quantified as internal controls, and each value represents mean ± Sd (Student's *t* test, *n* = 3, **P < 0.01, and ****P < 0.0001).

The ORF of *RchiOBHmChr1g0380691* is 639 bp long, encoding a protein of 213 amino acids. Phylogenetic analysis showed that RchiOBHmChr1g0380691 is most closely related to CBL4/SOS3 in *A. thaliana* (Supplemental Fig. S3); hence, this gene was named *RhCBL4*. To validate the expression pattern of *RhCBL4*, we performed reverse transcription quantitative PCR (RT-qPCR). *RhCBL4* was expressed at relatively low level from Stages 1 to 3 but at significantly higher levels at Stages 5 and 6 (Fig. 2A). Furthermore, *RhCBL4* expression was significantly induced by exogenous ethylene treatment (Fig. 2B). These results suggest that *RhCBL4* is a senescence-related gene that is induced by ethylene.

To further explore the role of RhCBL4 in petal senescence, we silenced *RhCBL4* expression in rose by virus-induced gene silencing (VIGS) using a tobacco rattle virus vector (TRV-*RhCBL4*) constructed from a 3′ terminal fragment (521 bp long) of *RhCBL4* cDNA. The transcript level of *RhCBL4* was significantly reduced in *RhCBL4*-silenced petals vs. the TRV controls (Fig. 2C). Under air conditions, the *RhCBL4*-silenced flowers displayed distinctly slower senescence than control plants (Fig. 2D). The life span of flower from opened bud to completed senescence was 9.8 ± 1.6 d for *RhCBL4*-silenced flowers, compared to 7.3 ± 0.9 d for TRV control flowers (Fig. 2E). After 10 ppm ethylene treatment for 12 h, flower senescence in both the control and silenced plants was accelerated, but life span of the silenced flowers (6.9 ± 1.2 d) was longer than of the controls (4.4 ± 0.7 d) (Fig. 2, D and E). We also evaluated the expression of *RhSAG12*, which was markedly lower in *RhCBL4*-silenced flowers than the control (Fig. 2F). These results indicate that RhCBL4 positively regulates petal senescence.

### RhCBL4 physically interacts with RhCIPK3

**Figure 3.**
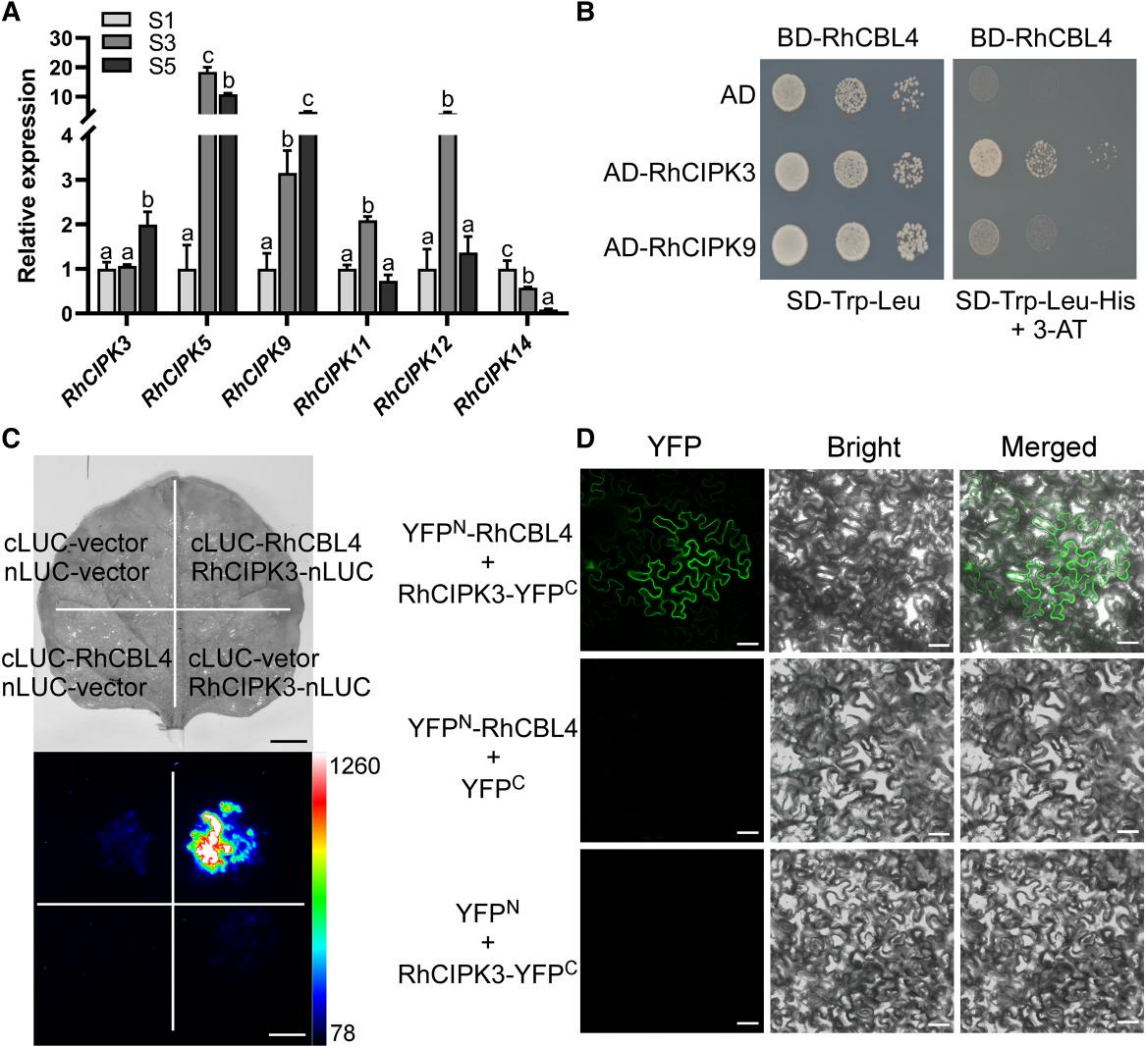
RhCBL4 interacts with RhCIPK3. **A)** Expression patterns of 6 *CIPK* family genes in the petal at different developmental stages of flower opening. *RheIF5A* and *RhUBI2* were quantified as internal controls (mean ± Sd, *n* = 3, *P* < 0.05, 1-way ANOVA). **B)** Interaction between RhCBL4 and RhCIPK3/9 in a Y2H assay. The pGADT7 (AD) empty vector serves as negative control. The yeast colonies were selected on the synthetic dropout (SD) medium -Trp/-Leu and -Trp/-Leu/-His with 5 mM 3-AT (3-amino-1,2,4-triazole). BD, pGBKT7 vector. **C)** Interaction between RhCBL4 and RhCIPK3 analyzed using Split-LUC complementation assay. RhCBL4-cLUC and RhCIPK3-nLUC were coinfiltrated into *N. benthamiana* leaves. The empty nLUC and cLUC constructs were used as negative controls. The pseudocolor bar represents the range of luminescence intensity in the image. Scale bar represents 1 cm. **D)** Interaction between RhCBL4 and RhCIPK3 analyzed using BiFC assays. The YFP^N^-RhCBL4 and RhCIPK3-YPF^C^ were co-transformed in *N. benthamiana* leaves. The empty YFP^N^ and YPF^C^ constructs were used as negative controls. Scale bar represents 50 *µ*m.

To identify RhCBL4-interacting proteins, we performed yeast 2-hybrid (Y2H) interaction screens using a cDNA prey library from rose petals. The screens identified 44 interactors (Supplemental Table S3). As expected, the screen yielded 8 CBL-interacting protein kinase (CIPK) proteins (Supplemental Table S3 and Fig. S4). Six of the 8 candidate *CIPK* genes were highly expressed in our petal transcriptome. We cross validated the expression patterns these 6 CIPK genes in rose petals. The expression of *RhCIPK3* and *RhCIPK9* increased at Stage 5 relative to Stage 3 (Fig. 3A). We also examined the expression of the 6 *CIPK* genes in response to ethylene treatment. Except for *RhCIPK3* and *RhCIPK9*, all other *CIPK* genes were significantly downregulated after ethylene treatment (Supplemental Fig. S5). To further test the interaction between RhCBL4 and RhCIPK3 or RhCIPK9, the full-length ORF of *RhCIPK3* and *RhCIPK9* was cloned into the pGADT7 vector and performed Y2H analysis. RhCBL4 interacted with RhCIPK3 in yeast, while RhCIPK9 did not directly interact with RhCBL4 (Fig. 3B). These results further support the interaction between RhCIPK3 and RhCBL4.

We validated this interaction by performing split-luciferase (Split-LUC) assays in *Nicotiana benthamiana*. We transferred combinations RhCIPK3-nLUC/cLUC-RhCBL4, nLUC/cLUC-RhCBL4, RhCIPK3-nLUC/cLUC, and nLUC/cLUC into *N. benthamiana* leaves. The signals were only detected in the combination of RhCIPK3-nLUC and cLUC-RhCBL4, whereas almost no LUC activity was detected when empty vectors were used (Fig. 3C). We also performed bimolecular fluorescence complementation (BiFC) assay to further evaluate the subcellular localization of the interaction between RhCIPK3 and RhCBL4. We coinfiltrated *N. benthamiana* leaves with *Agrobacterium tumefaciens* cells carrying the YNE173-*RhCBL4* and YCEm-*RhCIPK3* plasmids. In parallel, each fusion construct was cotransfected with empty vectors as control. The yellow fluorescent protein (YFP) signals were only detected when RhCBL4-YFP^N^ was coexpressed with RhCIPK3-YFP^C^. The signals were detected throughout the cells, including the cytoplasm, plasma membrane, and nucleus (Fig. 3D). By contrast, YFP fluorescence was undetectable in the negative controls. These results demonstrate that RhCBL4 physically interacts with RhCIPK3 in plant cells.

### Silencing *RhCIPK3* delays petal senescence

**Figure 4.**
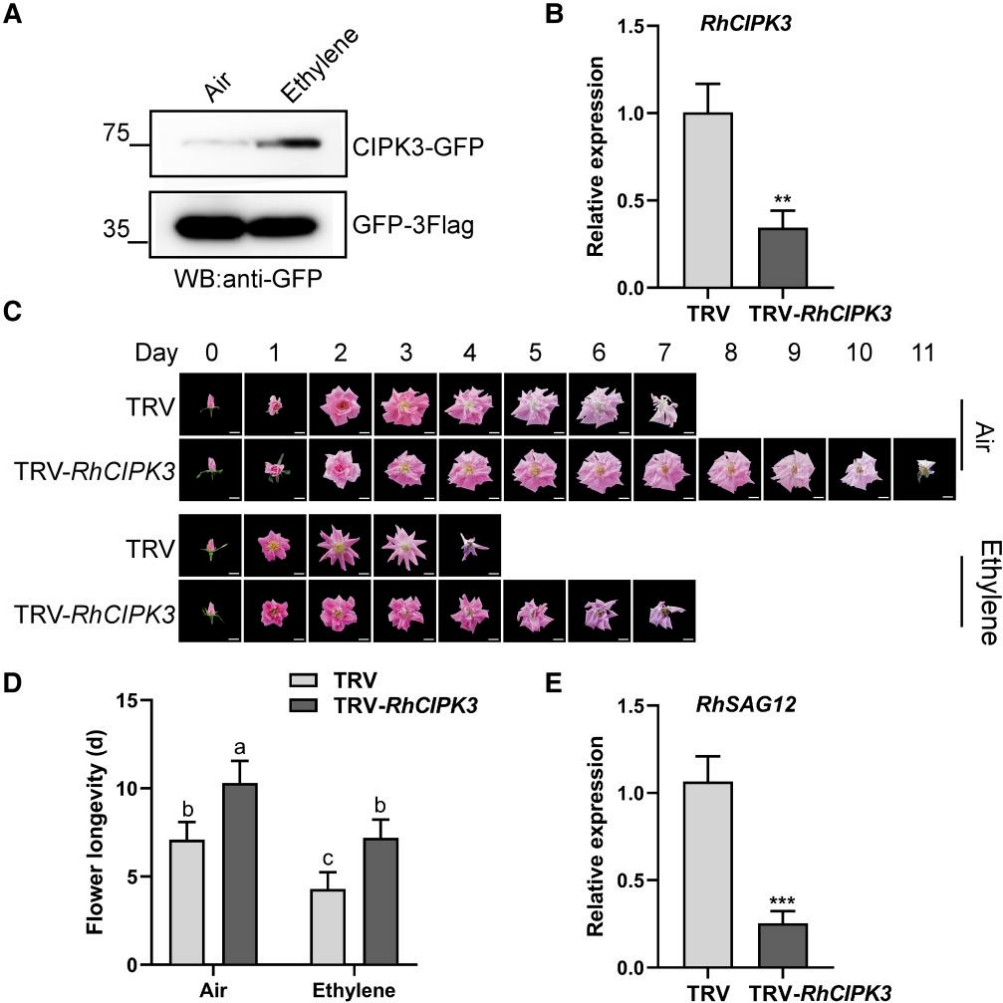
Silencing of *RhCIPK3* delays petal senescence in rose. **A)** Protein levels of RhCIPK3 after air or ethylene treatment for 12 h. Western blot (WB) analysis of total petal protein extracts using anti-GFP antibody. GFP-Flag was probed and served as a loading control. **B)** Expression of *RhCIPK3* in the petals of TRV control plants and *RhCIPK3*-silenced plants. *RheIF5A* and *RhUBI2* were quantified as internal control (mean ± Sd, *n* = 3, **P < 0.01, Student's *t* test). **C)** Phenotypes of TRV control and *RhCIPK3*-silenced flowers with air or ethylene treatment were recorded daily. Images were digitally extracted for comparison. Scale bar represents 2 cm. **D)** The duration of flower senescence in control and *RhCIPK3*-silenced flowers (mean ± Sd, *n* = 10, *P* < 0.05, 2-way ANOVA). **E)** Relative expression levels of *RhSAG12* in control and *RhCIPK3*-silenced petals by RT-qPCR. *RheIF5A* and *RhUBI2* were quantified as internal controls (mean ± Sd, *n* = 3, ***P < 0.001, Student's *t* test).

The gene expression of *RhCIPK3* has no significant alteration upon ethylene treatment. We reasoned that the regulation of RhCIPK3 might occur at the protein level and not at the transcriptional level. We first searched for CIPKs in our previous proteome data set from rose petals (Lu et al. 2019), finding that 4 CIPKs were detected by mass spectrometry (Supplemental Table S4). The protein level of RhCIPK3 increased by 5.5-fold from Stages 3 to 5 (Supplemental Table S4). To further investigate how ethylene influences RhCIPK3 protein in rose petal, we monitored the protein level of RhCIPK3 in response to ethylene treatment. RhCIPK3 protein levels obviously increased after 12 h of ethylene treatment (Fig. 4A).

To evaluate the biological role of RhCIPK3 during petal senescence, we constructed the TRV-*RhCIPK3* vector using a 3′ terminal region of *RhCIPK3* (390 bp long) to specifically silence *RhCIPK3*. The expression of *RhCIPK3* was significantly lower in *RhCIPK3*-silenced petals than that in TRV control petals (Fig. 4B). *RhCIPK3*-silenced flowers showed delayed petal senescence compared to TRV control flowers (Fig. 4C); these phenotypes are similar to those of *RhCBL4*-silenced flowers (Fig. 2D). The life span of *RhCIPK3*-silenced flowers (10.3 ± 1.3 d) was longer than that of control flowers (7.1 ± 1.0 d) (Fig. 4D). After ethylene treatment, life span of the silenced flowers (7.2 ± 1.0 d) was still longer than that of the TRV controls (4.3 ± 0.9 d) (Fig. 4D). And RT-qPCR showed that the expression of *RhSAG12* was significantly reduced in *RhCIPK3*-silenced petals vs. the TRV controls (Fig. 4E). These results indicate that RhCIPK3 also positively regulates ethylene-induced senescence in rose.

In addition, to understand whether the *CIPK3* gene has conserved role in angiosperm, we also observed the effect of ethylene on senescence of *atcipk3*, an *Arabidopsis* mutant. The results showed that after treatment of 100 *µ*M 1-aminocyclopropane-1-carboxylic acid (ACC), an ethylene precursor, compared with *Arabidopsis* wild-type plants, the leaves of *atcipk3* exhibited delayed leaf yellowing (Supplemental Fig. S6A), and chlorophyll contents were significantly higher in *atcipk3* leaves (Supplemental Fig. S6B). As expected, treatment of AgNO_3_ (silver nitrate), an ethylene action inhibitor, achieved an opposite effect as ethylene treatment in both phenotype and chlorophyll contents of leaves (Supplemental Fig. S6).

### RhCIPK3 interacts with RhJAZ5 in the nucleus

To identify possible interacting partners of RhCIPK3, we used RhCIPK3 as bait to screen for potential interacting proteins by Y2H screening. More than 20 positive colonies were obtained and identified by sequencing (Supplemental Table S5). One particularly interesting putative interacting partner, RchiOBHmChr2g0146371, is a JAZ protein that contains a ZIM/TIFY and a Jas motif (Supplemental Fig. S7) (Vanholme et al. 2007; Chung et al. 2009). We identified 11 JAZ genes from the *Rosa chinensis* genome data (Supplemental Fig. S7) (Raymond et al. 2018). RchiOBHmChr2g0146371 shares only 30% sequence identity with *Arabidopsis* JAZ proteins, while rose and strawberry belong to the *Rosaceae* and have a close genetic relationship (Yan, Byrne, et al. 2018). We therefore performed phylogenetic analysis of JAZ proteins from rose, strawberry, and *Arabidopsis* and renamed RchiOBHmChr2g0146371 as RhJAZ5 (Supplemental Fig. S8).

**Figure 5.**
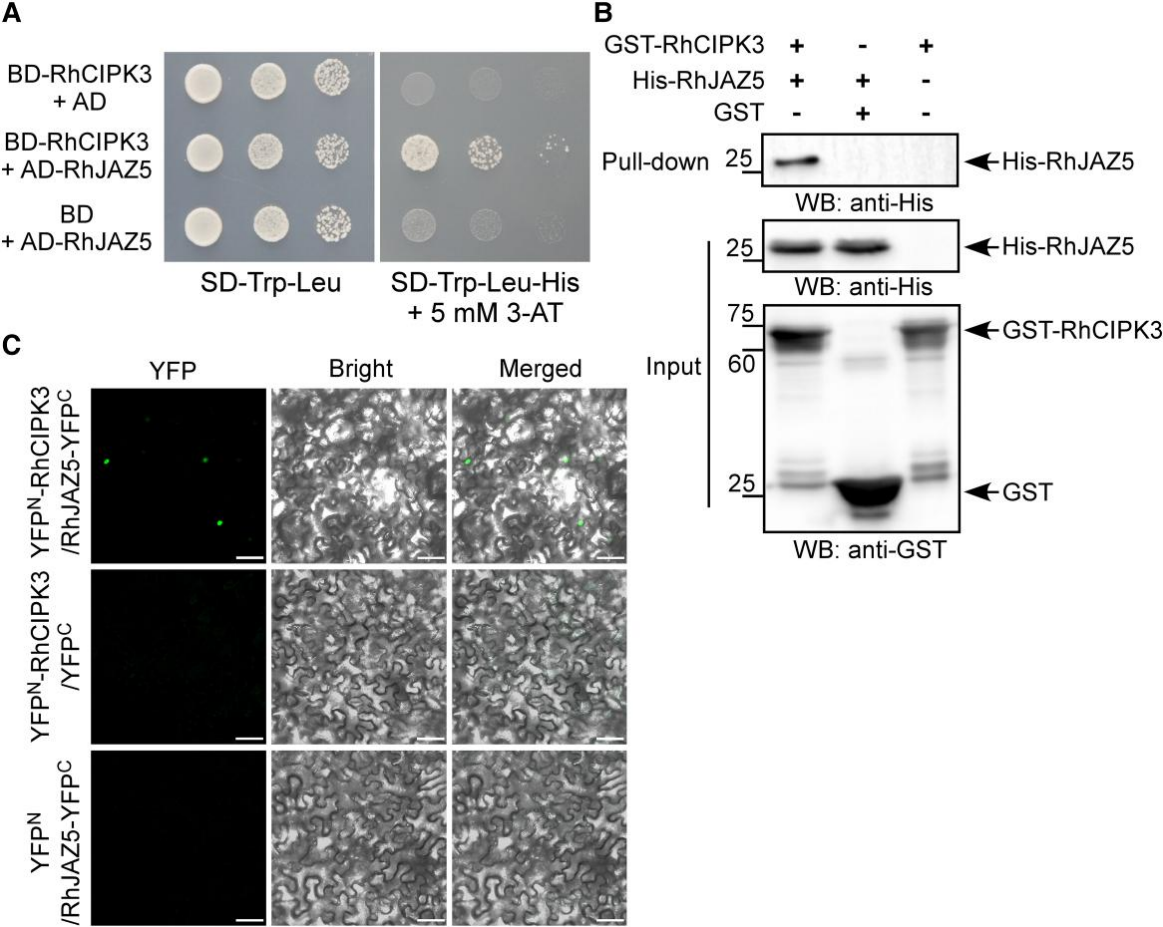
RhCIPK3 interacts with RhJAZ5. **A)** Interaction between RhCIPK3 and RhJAZ5 in a Y2H assay. The pGADT7 (AD) and pGBKT7 (BD) empty vectors serve as negative controls. The yeast colonies were selected on the synthetic dropout (SD) medium -Trp/-Leu and -Trp/-Leu/-His with 3-AT (3-amino-1, 2, 4-triazole). **B)** Interaction between RhCIPK3 and RhJAZ5 analyzed using in vitro pull-down assay. Purified GST-RhCIPK3 or GST proteins were incubated with His-RhJAZ5 and immunoprecipitated with GST beads. The interaction was detected by immunoblotting with anti-His antibody. WB, western blot. **C)** Interaction between RhCIPK3 and RhJAZ5 analyzed using BiFC assays. The YFP^N^-*RhCIPK3* and *RhJAZ5*-YPF^C^ were cotransformed in *N. benthamiana* leaves. The empty YFP^N^ and YPF^C^ constructs were used as negative controls. Scale bar represents 50 *µ*m.

To further validate the interaction of RhCIPK3 and RhJAZ5, we cloned the full-length ORF of RhJAZ5 into the prey vector and confirmed the interaction of this protein with RhCIPK3 by Y2H (Fig. 5A). We also tested for interactions between RhCIPK3 and 2 JAZ homologs, RhJAZ1 and RhJAZ4; however, RhCIPK3 failed to interact with these 2 JAZs in yeast (Supplemental Fig. S9A). We examined the interaction of RhCIPK3 and RhJAZ5 using an in vitro pull-down assay. The recombinant protein GST-RhCIPK3 was attached to GST-Sepharose beads to trap the His-RhJAZ5 prey protein. GST-RhCIPK3 effectively pulled down His-RhJAZ5, whereas GST protein did not (Fig. 5B). To determine whether RhCIPK3 and RhJAZ5 interact in plant cells, we performed BiFC by fusing full-length RhCIPK3 and RhJAZ5 to the N- and C-terminal region of YFP, respectively. Samples coinfiltrated with YFP^N^-*RhCIPK3* and *RhJAZ5*-YFP^C^ showed YFP signal in the nucleus, whereas all control samples yielded no signal (Fig. 5C). These results indicate that RhCIPK3 physically interacts with RhJAZ5 in the nucleus.

### Overexpression of RhJAZ5 delays petal senescence

In our rose transcriptome, 6 of 11 *JAZ*s were expressed in petals (Supplemental Fig. S9B). We further analyzed the expression patterns of these 6 *JAZ*s at different developmental stages by RT-qPCR. The expression of *RhJAZ5* is upregulated from Stages 1 to 3 and remains high in Stage 5, while the other *JAZ* genes tend to be repressed or their expression does not change during petal senescence (Supplemental Fig. S9B). These results suggest that *RhJAZ5* might play a unique role in petal senescence.

**Figure 6.**
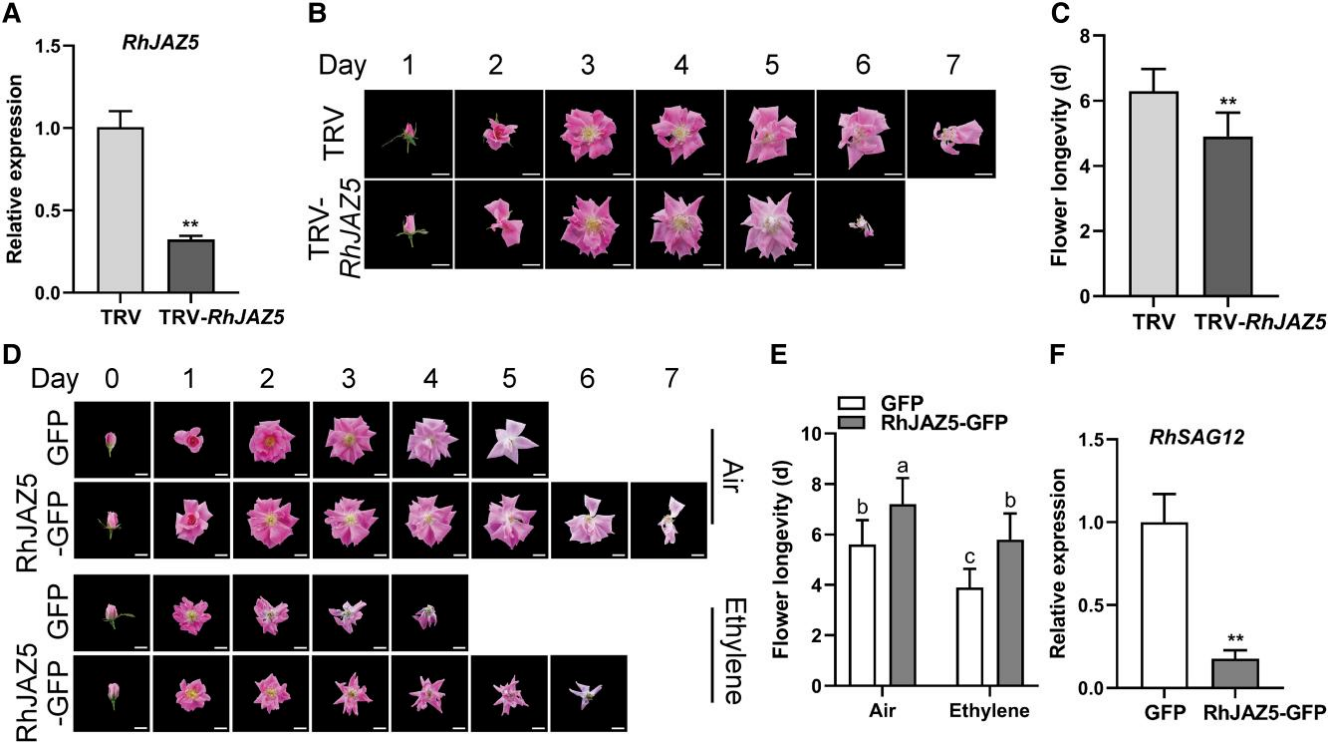
RhJAZ5 counteracts flower senescence in rose. **A)** Expression of *RhJAZ5* in the petals of TRV control plants and *RhJAZ5*-silenced plants (mean ± Sd, *n* = 3, **P < 0.01, Student's *t* test). **B)** Phenotypes of TRV control and *RhJAZ5*-silenced flowers were recorded daily. Scale bar represents 2 cm. **C)** The duration of flower senescence in control and *RhJAZ5*-silenced flowers (mean ± Sd, *n* = 10, **P < 0.01, Student's *t* test). **D)** Phenotypes of control and RhJAZ5 overexpression flowers with air or ethylene treatment were recorded daily. Images in **B)** and **D)** were digitally extracted for comparison. Scale bar represents 2 cm. **E)** The duration of flower senescence in control and RhJAZ5 overexpression flowers (mean ± Sd, *n* = 10, *P* < 0.05, 2-way ANOVA). **F)** Relative expression levels of *RhSAG12* in control and overexpressed RhJAZ5 petals by RT-qPCR. *RheIF5A* and *RhUBI2* were quantified as internal controls (mean ± Sd, *n* = 3, **P < 0.01, Student's *t* test).

To further explore the role of RhJAZ5 in petal senescence, we silenced *RhJAZ5* by VIGS (Fig. 6A). Compared to the control flowers, *RhJAZ5*-silenced flowers showed accelerated senescence (Fig. 6, B and C). We also transiently overexpressed *RhJAZ5* in rose flowers to generate RhJAZ5-OE flowers (Supplemental Fig. S10). In contrast to the silenced flowers, RhJAZ5-OE flowers showed delayed petal senescence (Fig. 6D). The life span of RhJAZ5-OE flowers (7.2 ± 1.0 d) was longer than that of control flowers (5.6 ± 1.0 d) (Fig. 6E). After ethylene treatment, life span of the overexpression flowers (5.8 ± 1.0 d) was still longer than that of the TRV control flowers (3.9 ± 0.7 d) (Fig. 6E). In addition, *RhSAG12* was expressed at lower level in RhJAZ5-OE petals than the control (Fig. 6F). These results suggest that RhJAZ5 represses ethylene-induced petal senescence.

### RhCIPK3 modulates the stability of RhJAZ5 protein

**Figure 7.**
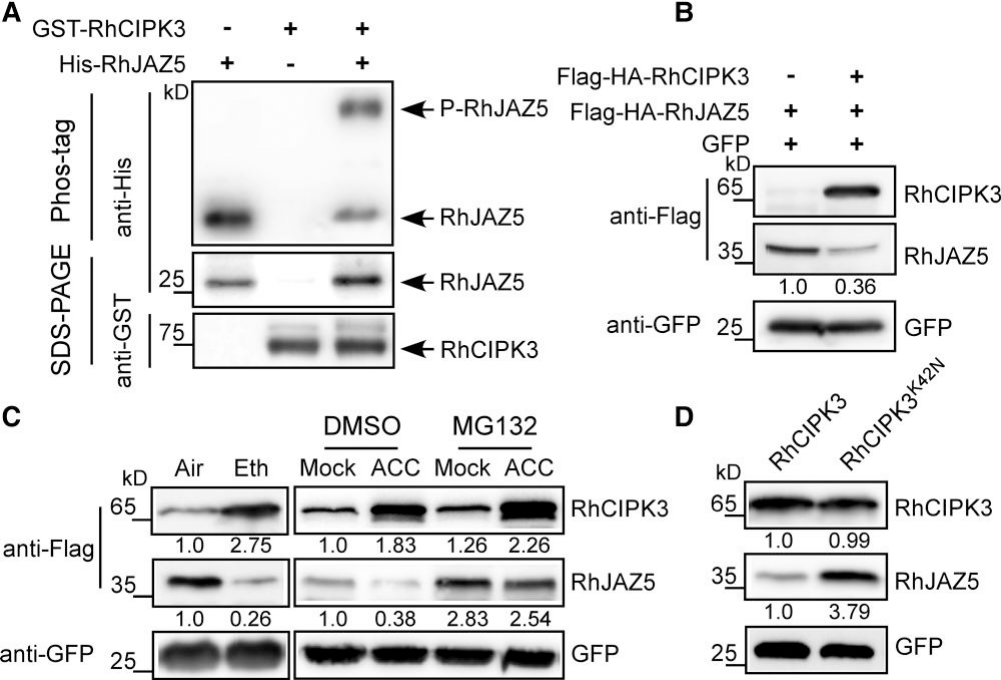
RhCIPK3 influences the stability of RhJAZ5. **A)** In vitro phosphorylation assay of RhJAZ5 by RhCIPK3. Phosphorylated RhJAZ5 (P-RhJAZ5) were analyzed by Phos-tag SDS–PAGE. **B)** The stability of RhJAZ5 protein after coexpression with RhCIPK3. Proteins were extracted from rose petals transiently expressing Flag-HA-RhJAZ5 alone or coexpressing with Flag-HA-RhCIPK3. RhJAZ5 and RhCIPK3 were detected by anti-Flag antibody. GFP was probed with GFP antibody and served as a loading control. **C)** Protein stability in the presence or absence of ethylene/ACC. Petals were transiently overexpressed RhJAZ5 and RhCIPK3 for 2 d and then treated with ethylene, or ACC with or without 50 *µ*M MG132 for 12 h. **D)** The effect of RhCIPK3 different variants on RhJAZ5 protein stability. Immunoblot analysis of RhJAZ5 accumulation after coexpressed with different RhCIPK3 kinase variants. For **B) to D)**, the numbers indicate the relative band intensities of RhJAZ5 and RhCIPK3 normalized to GFP. kD, kilo Dalton.

As RhCIPK3 is a protein kinase, the interaction between RhCIPK3 and RhJAZ5 prompted us to test whether RhCIPK3 could phosphorylate RhJAZ5. We purified GST-RhCIPK3 and His-RhJAZ5 proteins for an in vitro kinase assay by using a Phos-tag SDS–PAGE. A shifted band of RhJAZ5 was observed following incubation with RhCIPK3 (Fig. 7A). This result verifies that RhCIPK3 directly phosphorylates RhJAZ5.

Previous studies have shown that the phosphorylation status of JAZs affect their stability (Liu et al. 2017; He et al. 2020; Song et al. 2021). To investigate whether RhCIPK3 regulates the stability of RhJAZ5, we transiently transfected rose petals with constructs encoding Flag-tagged *RhJAZ5* (*35S*:3*Flag*-3*HA*-*RhJAZ5*) with or without Flag-tagged *RhCIPK3* (*35S*:3*Flag*-3*HA*-Rh*CIPK3*). Compared with single *RhCIPK3*-OE or *RhJAZ5*-OE, simultaneous overexpression of *RhCIPK3* and *RhJAZ5* did not get any additive effect on their expression levels (Supplemental Fig. S11). Immunoblot analysis showed that RhJAZ5 protein levels decreased when coexpressed with RhCIPK3 (Fig. 7B). Since ethylene treatment increased RhCIPK3 protein levels (Fig. 4A), we tested the effect of ethylene on the stability of RhJAZ5. Contrary to RhCIPK3, exogenous ethylene treatment promoted the degradation of RhJAZ5 protein (Fig. 7C). JAZs interact with the SCF complex and degrade through the 26S proteasome pathway (Chini et al. 2016; Yan, Yao, et al. 2018). We treated RhCIPK3 and RhJAZ5 cooverexpressed petals with MG132, a 26S proteasome inhibitor. Notably, MG132 treatment effectively blocked RhCIPK3-mediated degradation of RhJAZ5, regardless of whether the petals were treated with the ethylene precursor ACC (Fig. 7C), indicating that ethylene-induced RhCIPK3 promoted the degradation of RhJAZ5 via the 26S proteasome pathway.

We also examined whether the kinase activity of RhCIPK3 affects the protein stability of RhJAZ5. We first assessed the interaction of RhJAZ5 with the dead-kinase mutation of RhCIPK3 (RhCIPK3^K42N^), in which Kys^42^ was replaced by Asn (Liu et al. 2000). The kinase-dead RhCIPK3 mutation does not affect the RhCIPK3-RhJAZ5 interaction (Supplemental Fig. S12). Then, we detected the abundance of RhJAZ5 protein when coexpressed with RhCIPK3^K42N^. Compared to RhCIPK3, the use of RhCIPK3^K42N^ increased the abundance of RhJAZ5 protein (Fig. 7D). These results indicate that the phosphorylation of RhJAZ5 mediated by RhCIPK3 is required for its degradation process.

## Discussion

Petal senescence is a complex and irreversible process, which is regulated by external and internal cues (Rogers 2013; Ma et al. 2018). Calcium messengers play important roles in flower development, such as floral induction, floral organ differentiation, floral opening, and sexual reproduction (Wang et al. 2003; Ngo et al. 2014; Virdi et al. 2015; Nemoto et al. 2022). Cytosolic Ca^2+^ has long been known to be a common factor regulating senescence (van Doorn and Woltering 2008). However, the role for Ca^2+^ in petal senescence has remained elusive. Ethylene regulates the activity of Ca^2+^ channels at the transcriptional and posttranslational levels (Zhang et al. 2015). Therefore, we reasoned that calcium signaling might be involved in rose petal senescence and might be regulated by ethylene. Multiomic analyses showed that CBLs may be implicated in senescence (De Michele et al. 2009; Yun et al. 2012). In this study, we report that RhCBL4 plays a positive role in petal senescence and ethylene responses (Fig. 2). CBL4/SOS3 is vital for salt stress response (Ishitani et al. 2000; Qiu et al. 2002), yet its potential role in plant development has been largely overlooked.

CBL-CIPK networks are broadly involved in plant development and stress responses (Mao et al. 2022). The CBL-CIPK networks represent a distinct plant-specific paradigm for the decoding of Ca^2+^ signals (Tang et al. 2020). Here, we demonstrated that RhCBL4 physically interacts with RhCIPK3 during rose petal senescence (Fig. 3). However, an interaction between CBL4 and CIPK3 was not detected in *Arabidopsis* (Kim et al. 2000; Sanyal et al. 2017). AtCIPK3 was shown to interact with AtCBL1, AtCBL2, AtCBL3, and AtCBL9 (Pandey et al. 2008; Tang et al. 2015). CIPK3 serves as a negative regulator of ABA responses during seed germination (Kim et al. 2003) and is involved in transducing stress signals (Sanyal et al. 2017; Ju et al. 2022). *Arabidopsis* plants with knockout of *AtCIPK3* displayed delayed germination under ABA treatment and high Manganese (Mn) tolerance (Kim et al. 2003; Ju et al. 2022). Here, we found that CIPK3 has a conserved function in ethylene-induced senescence in both *Arabidopsis* and rose (Figs. 3 and S6).

In this study, we demonstrated that RhCIPK3 interacts with RhJAZ5 (Fig. 5). Several protein kinases have been reported to regulate JAZs. In *Malus domestica*, MdSnRK1.1 interacted with MdJAZ18 to regulate sucrose-induced anthocyanin biosynthesis (Liu et al. 2017). OsGSK2 binds to OsJAZ4 for enhancing plant antiviral defenses in rice (He et al. 2020). BIN2 interacts with JAZ proteins and negatively regulates plant defense in *Arabidopsis* and cotton (Song et al. 2021). In our study, we identified RhCIPK3 as an upstream kinase to phosphorylate JAZ protein. The kinase activity of RhCIPK3 is required for protein stability of RhJAZ5 but not for interaction (Figs. 7D and S12). However, it is unknown whether the interaction or phosphorylation of RhJAZ5 affects its ability to bind corepressor, such as NINJA and TOPLESS (Howe et al. 2018). And MG132 treatment abolished the effect of RhCIPK3-mediated degradation of RhJAZ5 protein, suggesting that the degradation is dependent on the 26S proteasome (Fig. 7C). In JA signal transduction, JAZ proteins are recruited by F-box protein COI1 for degradation by 26S proteasome, thereby releasing downstream JA response genes (Chini et al. 2016; Howe et al. 2018). In addition, CONSTANS (CO), a key photoperiod regulator, binds to JAZ3 and COI1 to promote flower senescence in *Arabidopsis* (Serrano-Bueno et al. 2022). It is possible that the degradation of RhJAZ5 is mediated by COI1. Nevertheless, COI1 mediates ethylene-induced root growth in a JA-independent manner (Adams and Turner 2010), and salicylic acid receptors are required for the degradation of JAZs in effector-triggered immunity (Liu et al. 2016). The specific molecular mechanism of RhJAZ5 degradation still needs to be studied in the future.

JAZ proteins are key repressors of JA signaling (Chini et al. 2007). However, the roles of JAZs in rose are still poorly understood. Here, we showed that RhJAZ5 is a unique rose JAZ gene whose expression remains at consistently high levels from Stages 3 to 5 of flower development (Supplemental Fig. S9B), revealing that RhJAZ5 plays a role in petal senescence. JAZs were involved in JA-induced leaf senescence (Yu et al. 2016; Tang et al. 2022), and JAZ3 is involved in flower senescence and abscission in *Arabidopsis* (Serrano-Bueno et al. 2022). Our results showed that *RhJAZ5* has an antagonistic effect on senescence in rose (Fig. 6). This antagonistic mechanism may be important for preventing premature senescence of flowers. JAZs can target diverse TFs from different families, such as basic helix–loop–helix (bHLH) and MYBs (Chini et al. 2016). JAZ4 and JAZ8 target and repress WRKY57, which regulates the key senescence genes (Jiang et al. 2014). MYC2, MYC3, and MYC4, as direct targets of JAZ repressors, activate JA-induced leaf senescence (Fernández-Calvo et al. 2011; Qi et al. 2015). JAZs bind and repress EIN3, which is a primary ethylene response gene (Zhu et al. 2011). EIN3 inhibits expression of microRNA164 and upregulates NAC2 expression in the regulation of leaf senescence (Li et al. 2013). However, whether RhJAZ5 regulates petal senescence through the genes mentioned above remains to be investigated.

Ethylene is the most documented phytohormone associated with flower senescence (Ma et al. 2018). Increasing ethylene levels during rose floral opening facilitates petal movement but accelerates petal senescence (Ma et al. 2005; Pei et al. 2013; Cheng et al. 2021). Phytohormones such as gibberellin (GA) and cytokinin (CTK) are reported act antagonistically to ethylene during rose flower senescence. RhHB1 mediates ethylene-accelerated flower senescence by inhibiting biosynthesis of GAs (Lü et al. 2014). RhHB6 and RhPR10.1 inhibit flower senescence by increasing the levels of CTKs (Wu et al. 2017; Khaskheli et al. 2018). JA has been shown in the regulation of floral senescence in *Arabidopsis* (Serrano-Bueno et al. 2022). JA-induced petal senescence is thought to stimulate ethylene production in orchid (Porat et al. 1995). Treatment with ethylene antagonist 1-methylcyclopropene (1-MCP) delays JA accelerated petal senescence process of rose (Zhang, Zhao, et al. 2019). And low levels of JA can rescue the ethylene response defect of the ethylene-insensitive mutant *ein2* during floral abscission (Kim et al. 2013). Since JAZs participate in plant growth and development and serve as hubs in the crosstalk between JA and other phytohormones (Kazan and Manners 2012), our findings here suggest that RhCIPK3 may link ethylene and JA signaling pathways through regulation of RhJAZ5 during rose petal senescence. Based on the results that silencing of *RhCBL4*/*RhCIPK3* and overexpression of *RhJAZ5* have the same effect and magnitude regardless of the ethylene treatment, the RhCBL4-RhCIPK3-RhJAZ5 module may be considered complementary to the canonical ethylene signal transduction pathway (ETRs-CTR1-EIN2-EIN3). Nevertheless, ethylene signaling and the RhCBL4-RhCIPK3-RhJAZ5 module may also be considered to operate in parallel. Therefore, more research is needed to clarify the underlying regulatory connection between our module and the ethylene pathway.

In conclusion, our data support a model in which under basal ethylene levels, RhJAZ5 inhibits the activities of downstream TFs that regulate senescence-related genes, thereby repressing senescence. Increased ethylene levels activate the RhCBL4-RhCIPK3 module, and then RhCIPK3 phosphorylates RhJAZ5 to promote its degradation through the 26S proteasome pathway, further inducing senescence (Supplemental Fig. S13). By recruiting CBL-CIPK to a phytohormonal regulatory pathway, plants regulate senescence in specific tissues and at specific times. Understanding these regulatory processes could help us to develop promising strategies to maximize flower longevity.

## Materials and methods

### Plant materials and growth conditions

Rose (*R. hybrida* cv. Samantha) plantlets, *N. benthamiana*, and *Arabidopsis* (*A. thaliana*) were grown in the substrate of peat soil: vermiculite = 2:1 at a greenhouse as described previously (Zhang, Wu, et al. 2021). The cuts of rose stem with 1 node were cultured in nutrient soil and grown in a growth chamber under controlled conditions (temperature of 23 ± 1 °C 16-/8-h light/dark cycle and relative humidity of 50% ± 10%). *A. thaliana* Col-0 was used as wild type. The *atcipk3* mutant is a SALK line (SALK_137779) as described previously (Ju et al. 2022).

### Ethylene treatment

Based on our previous observations of ethylene treatment (Ma et al. 2006), rose flowers were placed in a 40-L airtight container and treated with ethylene gas (final concentration 10 *μ*L L^−1^) for 12 h. Control flowers were treated with air. 1 M NaOH was placed in the container to prevent CO_2_ accumulation (Xue et al. 2008).

### Quantification of ethylene production

Ethylene measurements were performed as described previously (Ma et al. 2006). Each individual flower at different stages was collected and placed in an airtight container (35 mL). One-milliter head space gas of sample was withdrawn by using hypodermic syringe and then injected into the gas chromatograph (GC 17A, Shimadzu, Kyoto, Japan) for ethylene production measurement. Measurements were performed with 5 biological replicates.

### RNA isolation, RT-qPCR, and RNA-seq analysis

Total RNA was isolated from rose petal using the hot borate method as described previously (Zhang, Feng, et al. 2019). The cDNA was synthesized from 1-*µ*g RNA using HiScript III All-in one RT SuperMix Perfect for qPCR (Vazyme, China). RT-qPCR was performed using a ChamQ SYBR qPCR Master Mix (Vazyme, China) in an Applied Biosystems StepOnePlus real-time PCR system. The *RheIF5A and RhUBI2* genes were used as internal controls. The relative gene expression was calculated using the 2^−ΔΔCT^ method (Livak and Schmittgen 2001). The primers used for this assay are listed in Supplemental Table S6.

For RNA sequencing (RNA-seq) analysis, rose petals at different flower opening stages were collected in liquid nitrogen, and 3 biological replicates were generated. RNA-seq libraries were sequenced on an Illumina HiSeq 2500 system as described previously (Wu et al. 2022; Chen et al. 2023). The reads were mapped to the reference *R. chinensis* OldBlush database (https://lipm-browsers.toulouse.inra.fr/pub/RchiOBHm-V2), the rose transcriptome database (http://bioinfo.bti.cornell.edu/rose), and the *A. thaliana* database (https://www.arabidopsis.org). Use edgeR to analyze the difference between the genes assembled and quantized by StringTie (the threshold of significant difference is |log2[fold change]| ≥ 1, *P* [adj] < 0.05). Further determine the difference threshold according to the initial operation results. Meaning, adjust fold change and *P* value, and the genes meeting the threshold of significant difference are marked by the significant parameter. Raw sequencing data for all samples were deposited in the NCBI BioProject database under accession number PRJNA895937 (https://www.ncbi.nlm.nih.gov/bioproject/? term = PRJNA895937).

### VIGS

To silence the gene expression of *RhCBL4*, *RhCIPK3*, and *RhJAZ5* using the VIGS, gene-specific coding fragments were constructed to the tobacco rattle virus (pTRV2) vector as described previously (Zhang, Feng, et al. 2019; Liang et al. 2020). Briefly, *A. tumefaciens* GV3101 carrying derivatives of pTRV1, pTRV2, and pTRV2's was grown in liquid LB medium with selection antibiotics overnight. The transient transformation in rose plantlets was performed as described previously (Chen et al. 2021). The cells were collected and resuspended in the infiltration buffer (10 mM MES, 10 mM MgCl_2_, and 200 *µ*M acetosyringone, pH 5.8) to a final OD_600_ of 1.0. Rose plantlets were immersed in infiltration buffer with *Agrobacterium* containing pTRV1 and each recombinant pTRV2 and exposed to a vacuum of −25 kPa for 5 min twice. The plantlets were washed with distilled water and then placed in the dark at 8 °C for 3 d. The inoculated plants were transplanted into nutrient soil in the greenhouse. Three independent experiments were performed with at least 30 plantlets in each experiment. The flowers were photographed and recorded every day from Stage 1 until complete wilting (Ma et al. 2005). The duration of flower senescence was counted from the day of initial opening to the day of complete senescent stage. The primers used for this assay were designed as described in Supplemental Table S6.

### Phylogenetic analysis

The protein sequences of genes were first compared with ClusterW software and then compared with BioEdit software. The phylogenetic analyses were computed with MEGA 7.0 using the neighbor-joining algorithm in 1,000 replicates.

### Y2H assay

The full-length coding sequences of genes were cloned into the pGBKT7 (BD-) vector as bait and prey pGADT7 (AD-) vector as prey. The recombinant plasmids were transformed into the yeast strain *Saccharomyces cerevisiae* Y2HGold (Clontech). Yeast cotransformed with the empty pGADT7 vector was used as negative control. Transformed yeast colonies were selected on synthetic dropout medium minus Leu and Trp. Protein interactions were examined on selective medium (SD-Trp-Leu-His with 5 mM 3-amino-1,2,4-triazole) for 3 d at 30 °C. The primers used for this assay were designed as described in Supplemental Table S6.

### Split-LUC complementation assay

*RhCIPK3* and *RhCBL4* were constructed respectively into Nluc and Cluc vectors. The recombinant plasmids were transformed into *A. tumefaciens* GV3101. The *A. tumefaciens* strains were inoculated into LB (Rif + Kan) liquid medium and shaken overnight. The cells were collected and resuspended in infiltration buffer to a final OD_600_ of 1.0. The 2 kinds of GV3101 cells were mixed according to 1:1 volume ratio and injected into *N. benthamiana* leaves. After 36 to 48 h, the back of leaves was sprayed with 100 *µ*M fluorescein substrate D-luciferin, and the samples were kept in the dark for 5 min. The LUC signal was detected by plant living imaging system (Berthold LB985, Germany) with 5-min exposure time.

### BiFC assay

BiFC assays were performed as described previously (Nie et al. 2022). The ORFs of *RhCBL4*, *RhCIPK3*, and *RhJAZ5* were inserted into pSYNE (R) or pSYCEm plasmids to generate YFP^N^-*RhCBL4*, *RhCIPK3*-YFP^C^, YFP^N^-*RhCIPK3*, and *RhJAZ5*-YFP^C^, respectively. Recombinant vectors were transformed into *A. tumefaciens* GV3101 strain. The combined plasmids were transformed into *Agrobacterium* cells. The *Agrobacterium* cells were cultured, collected, and finally resuspended with infiltration solution, correspondingly injected into appropriate *N. benthamiana* leaves. Fluorescence signals were detected using Zeiss LSM 800/880 confocal microscope system. YFP was excited at 488 nm by an argon laser, and the emission was detected between 500 and 550 nm.

### Assay for ethylene-induced leaf senescence

The leaf senescence assay was performed as previously described (Li et al. 2013). The third and fourth rosette leaves of 4 wk old were detached and floated on 3 mL of 3 mM MES buffer (pH 5.8) supplemented with or without 100 *µ*M ACC for 3 d in dark. For AgNO_3_ plus ACC treatment, leaves were pretreated with 10 mM AgNO_3_ for 1 h, washed with MES buffer, and treated with 100 *µ*M ACC for 3 d in dark.

Chlorophyll content was measured as previously described (Li et al. 2015). Briefly, leaves were weighed and incubated in 80% (*v*/*v*) acetone overnight in darkness. Absorption measured at 665, 663, and 645 nm. The chlorophyll content was calculated: chlorophyll a = 12.7 × A_665_ − 2.69 × A_645_; chlorophyll b = 22.9 × A_645_ − 4.68 × A_663_; and total chlorophyll = (chlorophyll a + chlorophyll b) × V/W.

### Protein purification from *Escherichia coli*

To obtain purified RhCIPK3 and RhJAZ5 proteins, pGEX-6p-1-*RhCIPK3* and pET28a-*RhJAZ5* plasmids were transformed into *E. coli* strain Rosetta (DE3) and then induced by 0.4 mM isopropyl β-D-thiogalactoside at 16 °C for 12 h. The recombinant protein was purified using glutathione agarose and Ni Sepharose according to the manufacturer's protocol (GE). The purified proteins were used in pull-down and in vitro protein kinase assays.

### In vitro pull-down assay

For pull-down assay, 0.5 *μ*g of His-RhJAZ5 proteins was incubated with 5 *μ*g of GST-RhCIPK3, and immunoprecipitated by GST beads at 4 °C for 2 h in pull-down buffer (PBS buffer containing 0.1% NP-40). After binding, beads was washed with PBS buffer for 4 to 5 times and boiled them as samples. The proteins were separated using 12% (*w*/*v*) SDS–PAGE. Anti-GST and anti-His antibodies were used to detect GST-RhCIPK3 and His-RhJAZ5, respectively.

### Transient overexpression in rose petal

For phenotype analysis, the transient transformation in rose flowers was performed as described previously (Liang et al. 2020). Briefly, the ORF sequence of *RhJAZ5* was constructed into Super1300 vector to study the overexpression of RhJAZ5 in rose petals. The vector was transformed into *A. tumefaciens* GV3101. The flowers at Stages 1 or 2 were immersed in the *Agrobacterium* suspension (OD_600_ = 1.0) and infiltrated under a vacuum at 0.7 MPa. The flowers were photographed and recorded every day until complete wilting.

For protein overexpression in rose petals, the outer whorl petals from Stage 2 flowers were injected with *Agrobacterium* suspension (OD_600_ = 0.5 to 1.0). Samples were harvested after 3 d of infiltration. For ethylene treatment, after 2 d of infection, flowers were treated with air or ethylene in airtight container for 12 h. For ACC and MG132 treatments, after 2 d of infection, petals were injected with 50 *μ*M ACC with or without 50 *μ*M MG132 for 12 h under room temperature. DMSO solution was used as mock control of MG132. Sampled petals were flash-frozen in liquid nitrogen and stored at −80 °C for protein extraction.

### In vitro kinase assay

All recombinant proteins were expressed in *E. coli* strain Rosetta and purified according to the manufacturer's protocol. One-microgram GST-RhCIPK3 was incubated with 2-*µ*g His-RhJAZ5 in kinase buffer (20 mM Tris–HCl, pH 8.0, 10 mM MgCl_2_, 100 *μ*M ATP, and 1 mM DTT) at 30 °C for 60 min. Reactions were terminated by the addition of SDS loading buffer at 95 °C for 10 min. The proteins were separated by 12% (*w*/*v*) SDS–PAGE or 8% Phos-tag SDS–PAGE (50 *µ*M Phos-tag Acrylamide). Anti-GST and anti-His antibodies were used to detect the proteins.

### Statistical analysis

All experiments were performed with at least 3 biological replicates. Statistical analysis was performed using GraphPad Prism version 8.0. All experimental data were tested with using Student's *t* test and 1-way or 2-way ANOVA as described in the corresponding figure legends. Asterisks indicate significant differences (*P < 0.05, **P < 0.01, ***P < 0.001, and ****P < 0.0001), and different letters indicate significant differences between groups (*P* < 0.05).

### Accession numbers

The GenBank (http://www.ncbi.nlm.nih.gov) accession numbers for rose genes used in this study are as follows: *RhSAG12* (*RchiOBHmChr5g0010401*), *RhNAC029* (*RchiOBHmChr4g0439451*), *RhNAC083* (*RchiOBHmChr4g0429461*), *RhNAC092* (*RchiOBHmChr2g0167461*), *RhWRKY22* (*RchiOBHmChr1g0357671*), *RhWRKY42* (*RchiOBHmChr5g0002561*), *RhCBL4* (*RchiOBHmChr1g0380691*), *RhCIPK1* (*RchiOBHmChr4g0427571*), *RhCIPK3* (*RchiOBHmChr5g0050061*), *RhCIPK5* (*RchiOBHmChr6g0299521*), *RhCIPK9* (*RchiOBHmChr1g0360871*), RhCIPK11 (*RchiOBHmChr5g0072521*), *RhCIPK12* (*RchiOBHmChr5g0011071*), *RhCIPK14* (*RchiOBHmChr3g0460881*), *RhCIPK20* (*RchiOBHmChr5g0011061*), *RhJAZ1* (*RchiOBHmChr2g0095481*), *RhJAZ4* (*RchiOBHmChr4g0443341*), *RhJAZ5* (*RchiOBHmChr2g0146371*), *RhJAZ7.2* (*RchiOBHmChr4g0387691*), *RhJAZ11* (*RchiOBHmChr4g0429271*), *RhJAZ12* (*RchiOBHmChr2g0096551*), *RheIF5A* (*RchiOBHmChr2g0085721*), and *RhUBI2* (*RchiOBHmChr1g0359561*).

## Supplementary Material

kiad365_Supplementary_Data

## Data Availability

All data generated during this study are included in this article and the Supplemental data. RNA-seq data that support the findings of this study have been deposited in the NCBI Bioproject database under accession number PRJNA895937.

## Supplemental data

The following materials are available in the online version of this article.

**Supplemental Figure S1.** Different stages and ethylene content of flowers during floral opening and senescence in rose (*R. hybrida* Samantha).

**Supplemental Figure S2.** RNA-seq analysis of rose petals during senescence.

**Supplemental Figure S3.** Phylogenetic analysis of rose and *Arabidopsis* CBL family proteins.

**Supplemental Figure S4.** Phylogenetic analysis of rose and *Arabidopsis* CIPK family proteins.

**Supplemental Figure S5.** Expression patterns of *CIPK* family genes in petals under ethylene treatment.

**Supplemental Figure S6.** *Arabidopsis atcipk3* mutant delays ethylene-induced leaf senescence.

**Supplemental Figure S7.** Multiple sequence alignment of JAZ family proteins in rose.

**Supplemental Figure S8.** Phylogenetic analysis of JAZs in different plants.

**Supplemental Figure S9.** RhCIPK3 does not interact with RhJAZ1 or RhJAZ4.

**Supplemental Figure S10.** Detection of RhJAZ5 in rose overexpression in petals.

**Supplemental Figure S11.** Relative expression of *RhJAZ5* and *RhCIPK3* in the 35S empty control petals and overexpression petals.

**Supplemental Figure S12.** The kinase activity of RhCIPK3 does not affect its interaction with RhJAZ5.

**Supplemental Figure S13.** A proposed model illustrating the role of the RhCBL4-RhCIPK3 module in regulating ethylene-induced petal senescence.

**Supplemental Table S1.** Upregulated DEGs at different stages and with ethylene treatment.

**Supplemental Table S2.** Identification of common upregulated DEGs.

**Supplemental Table S3.** Summary of Y2H screen of RhCBL4.

**Supplemental Table S4.** Proteins identified in petals (Stage 5 vs. Stage 3).

**Supplemental Table S5.** Summary of Y2H screen of RhCIPK3.

**Supplemental Table S6.** List of primers used in this study.
