# Supplementary material for: The CALCINEURIN B-LIKE 4/CBL-INTERACTING PROTEIN 3 module degrades repressor JAZ5 during rose petal senescence
Source: Plant Physiol. 2023 Jul 4;193(2):1605–20. doi: 10.1093/plphys/kiad365 (PMC10517193; doi:10.1093/plphys/kiad365)
Supplement: kiad365_Supplementary_Data [file kiad365_supplementary_data.zip › PP2023RA00245R2_Supplemental_Figures_1_13.pdf]

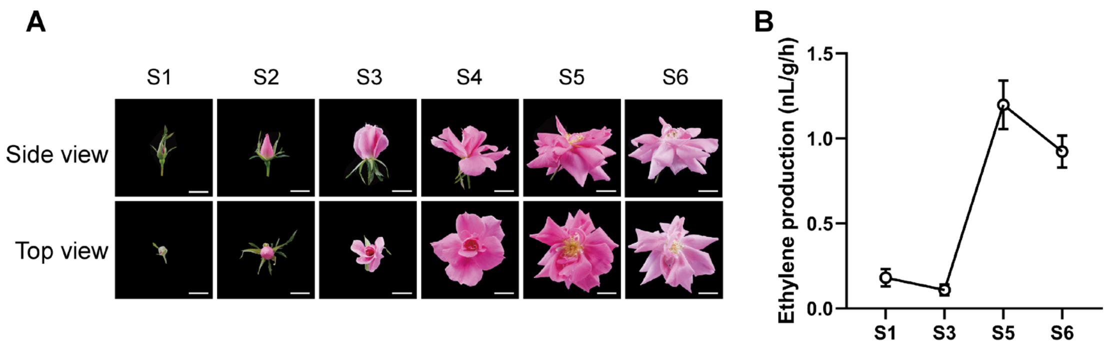


**Supplemental Figure S1. Different stages and ethylene content of flowers during floral opening and senescence in rose (*Rosa hybrida* Samantha).** (**A**) Different flower developmental stages of rose flowers. Stage 1 (S1), bud with partially visible petals; stage 2 (S2), partially opened flower bud; stage 3 (S3), flower with loose outer petals; stage 4 (S4), half-open flower (no anthers visible); stage 5 (S5), full opened flower (with visible anthers); stage 6 (S6), full opened flower with the occurrence of fading (senescence). Images were digitally extracted for comparison. Scale bar, 2 cm. (**B**) Ethylene production of rose flowers at different stages (mean ± SD, $n$ = 4).

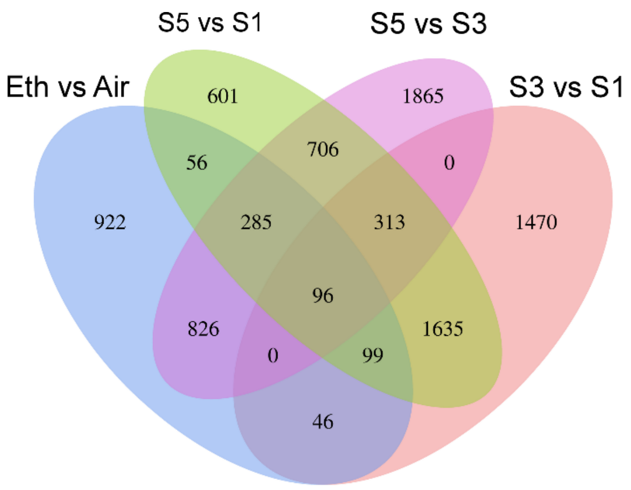


**Supplemental Figure S2. RNA sequencing analysis of rose petals during senescence.**

Venn diagram illustrating the distribution of differentially expressed genes (DEGs) between each two groups and the number of overlapping expression of genes. S, stage; Eth, ethylene. The DEGs were identified based on fold change [FC] ≥ 2 and false discovery rate [FDR] < 0.05.

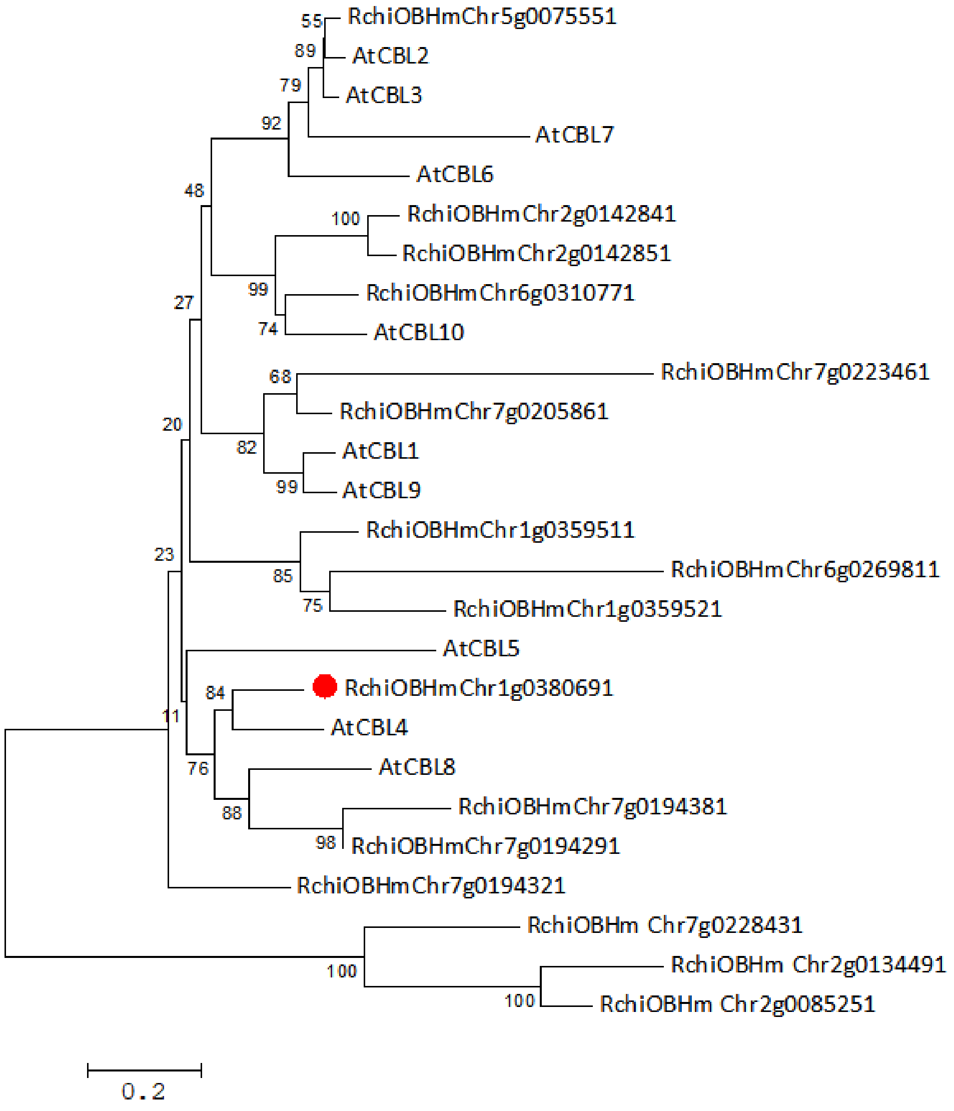


**Supplemental Figure S3. Phylogenetic analysis of rose and Arabidopsis CBL family proteins.**

Phylogenetic tree showing the evolutionary relatedness of *Rosa chinensis* OldBlush CBLs with that of *Arabidopsis*. The phylogenetic tree was constructed using MEGA 7.0 by the neighbor-joining method with amino acid p-distances and 1000 bootstrap replicates. The numbers on the branches indicate bootstrap percentages, and the scale bar indicates the nucleotide substitutions per site. The RhCBL4 protein analyzed in this study are marked with red closed circles.

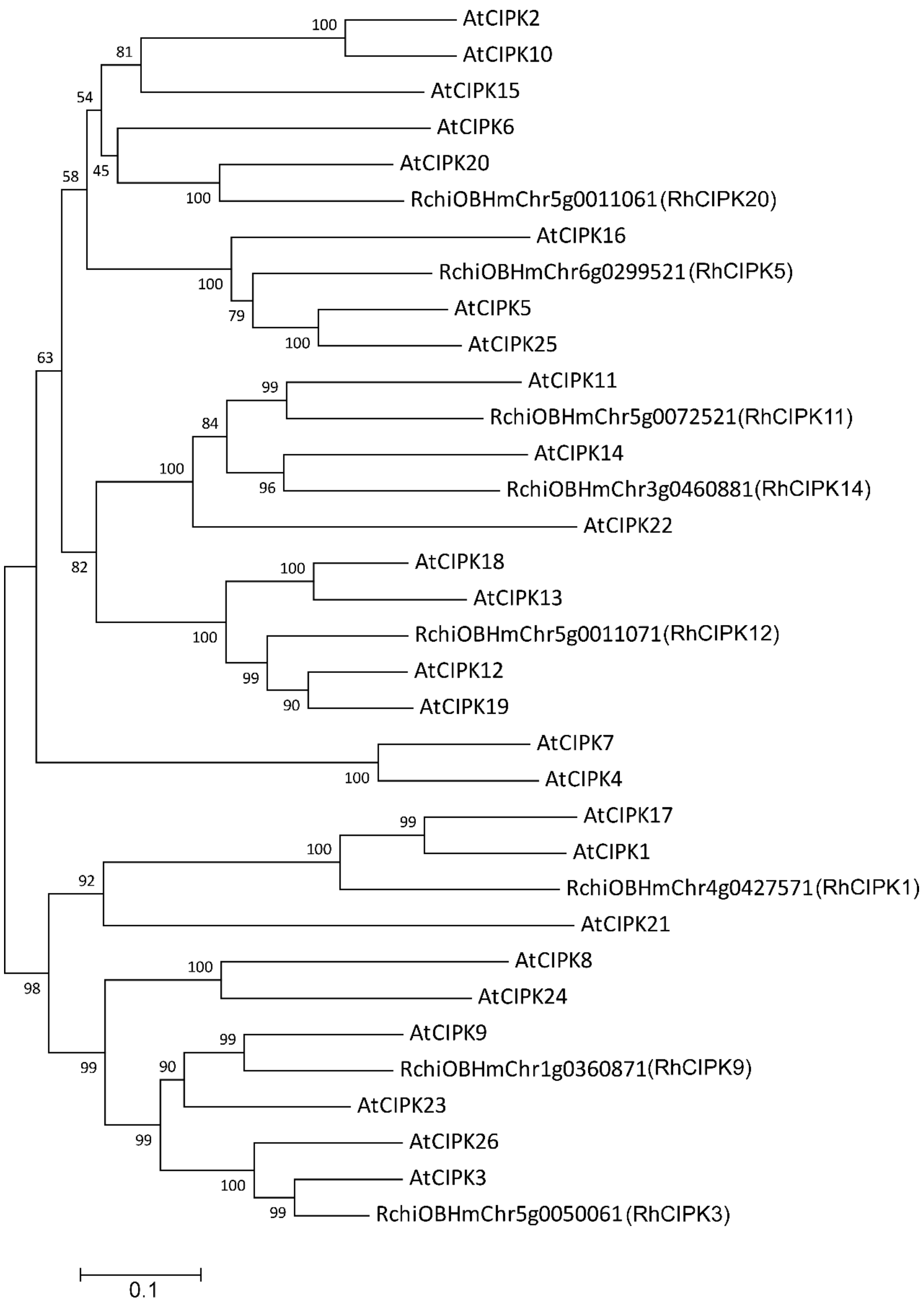


**Supplemental Figure S4. Phylogenetic analysis of rose and Arabidopsis CIPK family proteins.**

Phylogenetic tree showing the evolutionary relatedness of *Rosa chinensis* OldBlush CIPKs with that of *Arabidopsis*. The tree was constructed by the neighbour-joining method with MEGA program (v.7.0). Branch numbers represent as percentage of bootstrap values in 1000 sampling replicates and scale indicates branch lengths. The scale bar indicates the nucleotide substitutions per site.

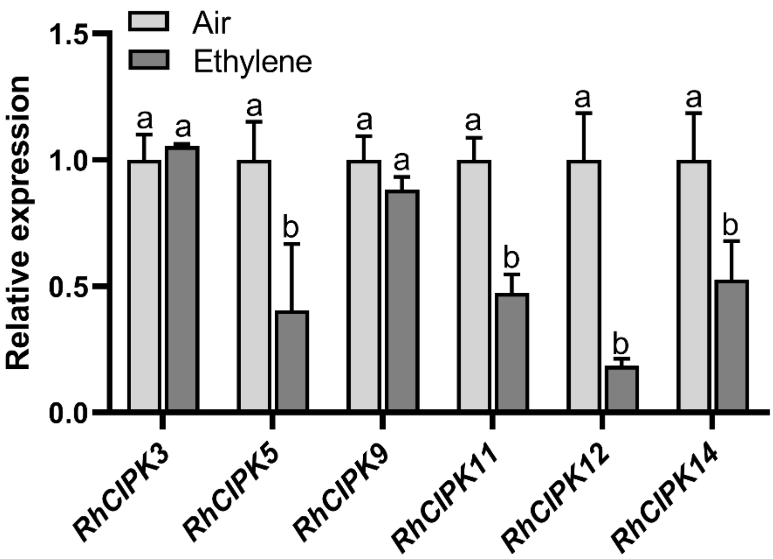


**Supplemental Figure S5. Expression patterns of *CIPK* family genes in petals under ethylene treatment.**

Expression of *CIPKs* transcript level in petal under 12 h ethylene treatment. *RheIF5A* and *RhUBI2* were quantified as internal controls (mean ± SD, $n = 3$, $P < 0.05$, Student's $t$ test).

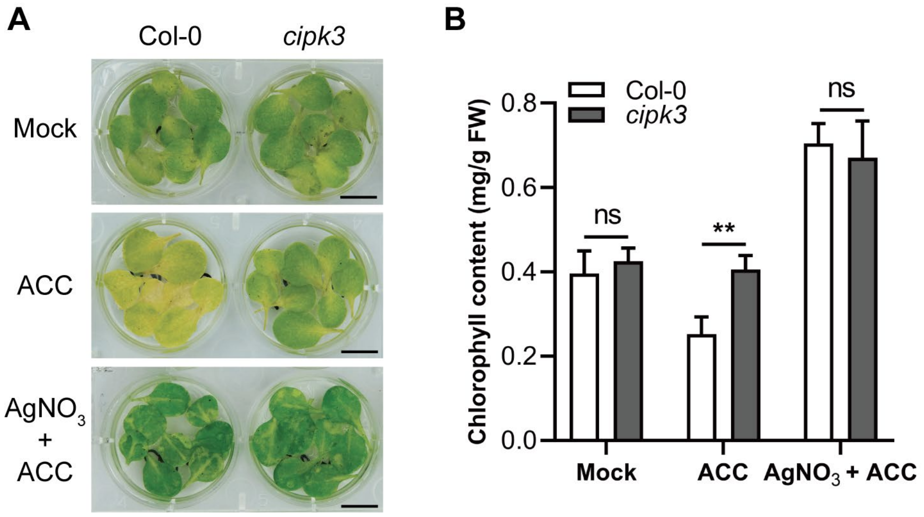


**Supplemental Figure S6. Arabidopsis *atcipk3* mutant delays ethylene-induced leaf senescence.**

(**A**) The senescence phenotypes of detached leaves of Col-0 and *atcipk3* (SALK_137779) plants treated with ACC (1-aminocyclopropane-1-carboxylic acid) and/or $AgNO_3$. Detached leaves were treated with MES (2-morpholinoethanesulphonic acid) buffer (Mock) and ACC for 3 d in dark. For $AgNO_3$ treatment, leaves were pretreated with $AgNO_3$ for 1 h, and treated with ACC for 3 d in dark. Scale bar represents 1 cm. (**B**) Chlorophyll contents in leaves from (A) were measured (mean ± SD, $n$ = 3, $^{**}P < 0.01$, Student's $t$ test). FW, fresh weight.

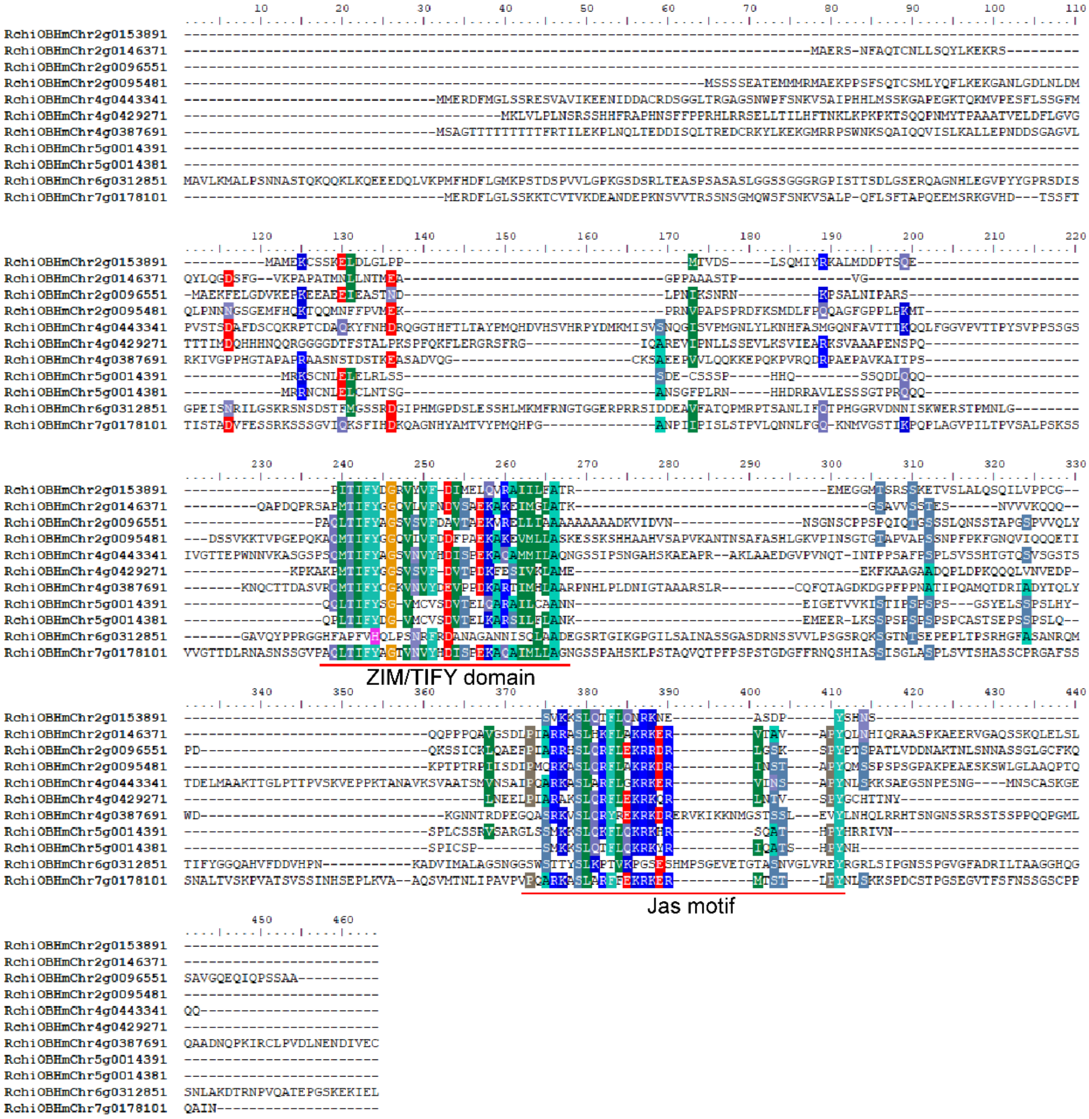


**Supplemental Figure S7. Multiple sequence alignment of JAZ family proteins in rose.**

Multiple sequence alignment of the deduced amino acid sequences of JAZs from *Rosa chinensis* OldBlush was conducted using BioEdit software. The numbers indicate amino-acid position, and identical amino acids are boxed in colors. Red bars indicate the conserved domain.

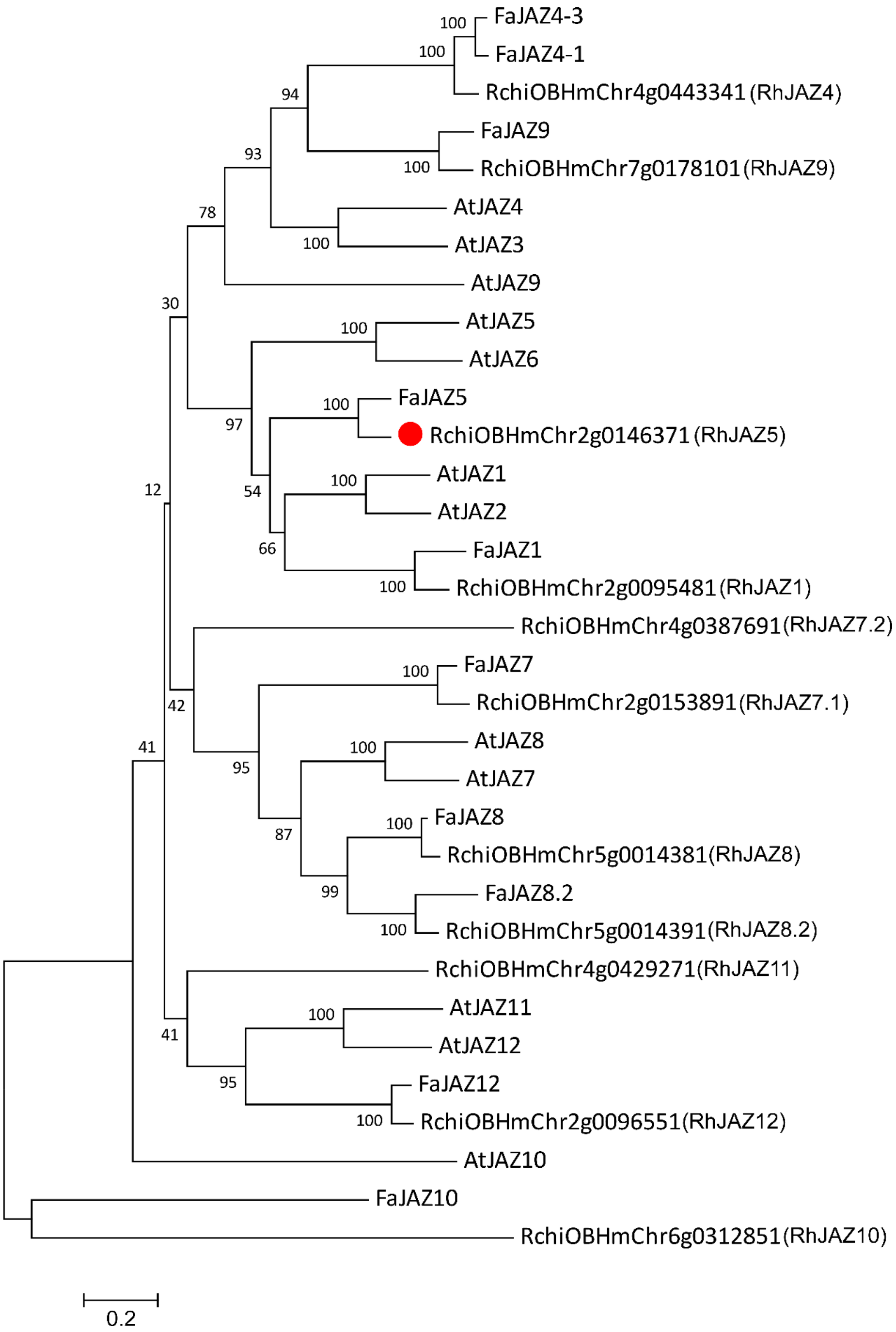


**Supplemental Figure S8. Phylogenetic analysis of JAZs in different plants.** Phylogenetic tree showing the evolutionary relatedness of *Rosa chinensis* OldBlush JAZs with that of other plants. The phylogenetic tree was constructed using MEGA 7.0 by the neighbor-joining method. Numbers along the branches indicate bootstrap support determined from 1000 repetitions, and the bar indicates an evolutionary distance of 0.2%. At, *Arabisdopsis thaliana*; Rh, *Rosa hybrida*; Fa, *Fragaria ananassa*.

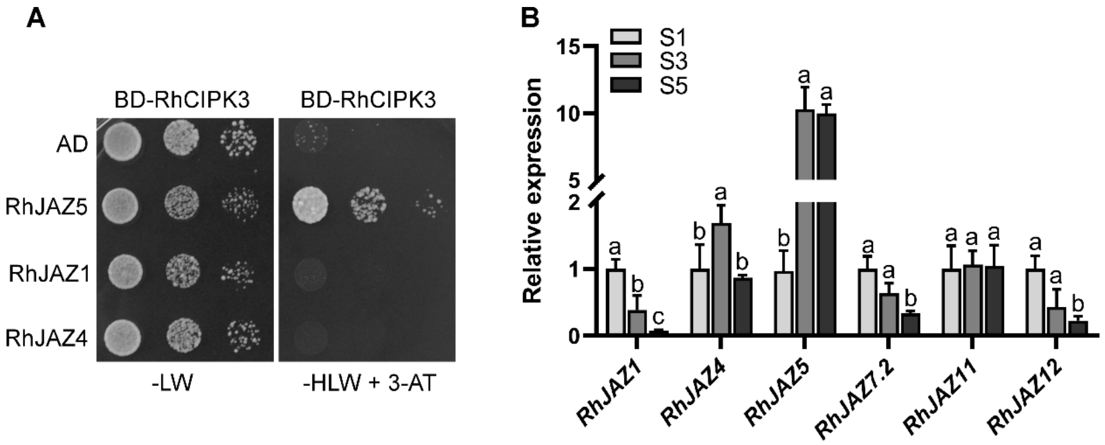


**Supplemental Figure S9. RhCIPK3 does not interact with RhJAZ1 or RhJAZ4.**

(**A**) Interaction between RhCIPK3 and RhJAZs in a yeast two-hybrid assay. The pGADT7 (AD) empty vector was served as negative control. The yeast colonies were selected on the synthetic dropout (SD) medium -Trp/-Leu and -Trp/-Leu/-His with 5 mM 3-AT (3-amino-1, 2, 4-triazole). BD, pGBKT7 vector. (**B**) Expression patterns of six *JAZ* family genes in the petal at different developmental stages of flower opening. *RheIF5A* and *RhUBI2* were quantified as internal controls (mean ± SD, $n$ = 3, $P < 0.05$, one-way ANOVA).

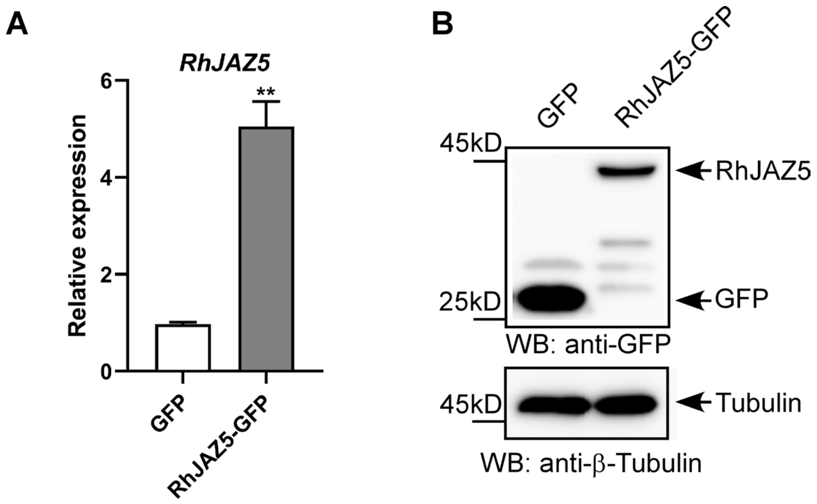


**Supplemental Figure S10. Detection of RhJAZ5 in rose overexpression in petals.**

(**A**) Relative expression levels of *RhJAZ5* in *Super*:*GFP* and *Super*:*RhJAZ5-GFP* overexpression petals by RT-qPCR. *RheIF5A* and *RhUBI2* were quantified as internal controls (mean ± SD, $n$ = 3, $^{**}P$ < 0.01, Student's $t$ test). (**B**) Validation of RhJAZ5 overexpression in petal cells by western blot (WB) analysis. Tubulin was used as a loading control. kD, kilo Dalton.

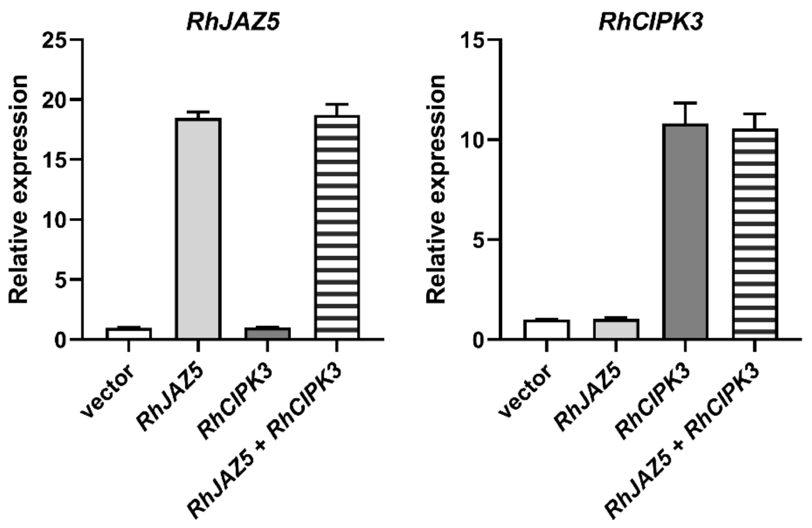


**Supplemental Figure S11. Relative expression of *RhJAZ5* and *RhCIPK3* in the 35S empty control petals and overexpression petals.**

Relative expression levels of *RhJAZ5* and *RhCIPK3* in empty vector, *35S*:*RhJAZ5*, *35S*:*RhCIPK3* solitary overexpression and co-overexpression petals by RT-qPCR. *RheIF5A* and *RhUBI2* were quantified as internal controls. Data represent means ± SD of three reproducible experiments.

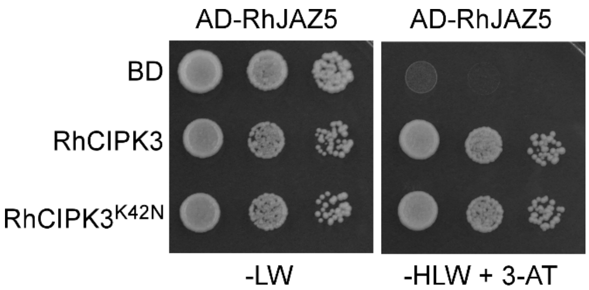


**Supplemental Figure S12. The kinase activity of RhCIPK3 does not affect its interaction with RhJAZ5.**

Interaction between RhCIPK3 and RhJAZ5 in a yeast two-hybrid assay. The pGBKT7 (BD) empty vector was served as negative control. RhCIPK3$^{K42N}$ is a kinase-dead mutation of RhCIPK3. AD, pGADT7 vector. The yeast colonies were selected on the synthetic dropout (SD) medium -Trp/-Leu and -Trp/-Leu/-His with 5 mM 3-AT (3-amino-1, 2, 4-triazole).

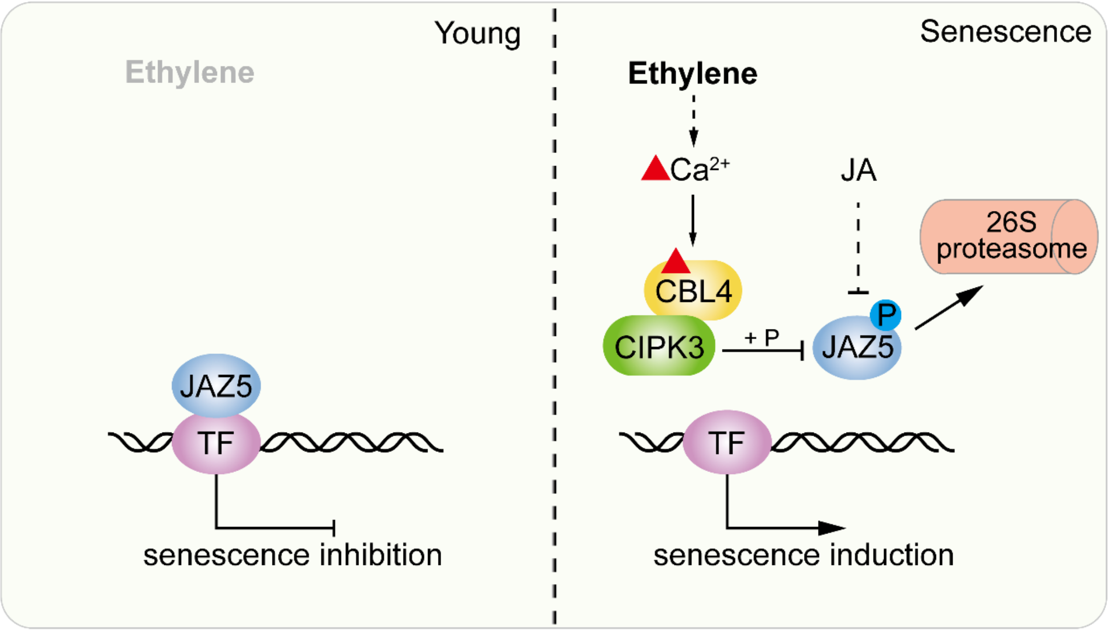


**Supplemental Figure S13. A proposed model illustrating the role of the RhCBL4-RhCIPK3 module in regulating ethylene-induced petal senescence.**

When ethylene is at low levels (early stages of flower opening), JAZ5 inhibits the activities of transcription factors that regulate senescence-related gene expression. Following flower opening and senescence, $Ca^{2+}$ and CBL4 production is induced by increasing ethylene levels. CBL4 interacts with CIPK3 to destabilize JAZ5, which further activates the senescence program. TF, transcription factor; JA, jasmonic acid; P, phosphorylation.
